# Dynamic breathing behaviour of the titanium-based metal–organic framework NTU-9 upon adsorption of water and organic solvents†

**DOI:** 10.1039/d5sc02585k

**Published:** 2025-06-25

**Authors:** Julia E. Knapp, Borja Ortín-Rubio, Fabian Heck, Kristina Gjorgjevikj, Anastasia Sleptsova, Simon Krause, Sebastian Bette, Bettina V. Lotsch

**Affiliations:** a Max Planck Institute for Solid State Research Heisenbergstraße 1 Stuttgart 70569 Germany s.bette@fkf.mpg.de b.lotsch@fkf.mpg.de; b Department of Chemistry, University of Stuttgart Pfaffenwaldring 55 Stuttgart 70569 Germany; c Department of Chemistry, University of Munich Butenandtstraße 5-13 Munich 81377 Germany

## Abstract

Understanding the structural response of framework materials to external stimuli has been of great interest. However, little is known about the stimuli responsiveness of titanium-based metal–organic frameworks. We investigate the reproducibility of the synthesis of the two-dimensional metal–organic framework NTU-9 (NTU = Nanyang Technological University) composed of Ti^4+^ cations and 2,5-dihydroxyterephtalate as the organic linker and the flexible response of the framework structure to external stimuli. Using acetic acid simultaneously as mediator and solvent leads to the reproducible formation of large NTU-9 crystallites after long reaction times. The MOF synthesis in isopropanol:acetonitrile (*i*-PrOH : MeCN) mixtures without a modulator is significantly faster but yields smaller NTU-9 crystallites. Pure heat treatment under ambient conditions removes a significant amount of the incorporated host solvent molecules without alteration of the framework's structure. After applying additional external stimuli (*i.e.*, vacuum), the material exhibits a pore distortion by compression in the lateral dimension, depending on the synthetic procedure. The new, distorted, metastable form NTU-9-d shows a reduction in unit cell volume, pore size, and crystal symmetry. Under humidity/air exposure or solvent resuspension, the framework reverts into its original state. The synthesis conditions significantly affect the flexibility of the MOF structure, where samples synthesized without modulator showed a lower tendency for distortion. Our results emphasise the importance of an in-depth understanding of the structure–property relationships in flexible MOFs through a detailed characterisation of the material's stimuli responsiveness process.

## Abstract 2

Synthesis optimisation and a reproducibility study of the titanium-based MOF NTU-9 with a detailed (*in situ*) characterisation lead to the discovery of its flexibility upon external stimuli (*e.g.*, vacuum) and the new distorted form NTU-9-d.

## Graphical abstract

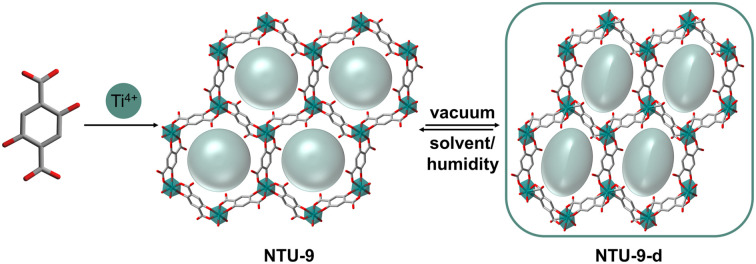

## Introduction

Metal–organic frameworks (MOFs) are porous reticular materials consisting of inorganic nodes (*e.g.*, metal ions, clusters) linked by organic bridging ligands through coordination bonds.^1–3^ Among them, MOFs constructed from tri- and tetravalent metal-based clusters combined with carboxylate-based ligands show higher stability according to the hard and soft acids and bases (HSAB) principle,^4^ which makes them suitable for applications such as gas storage and separation, as well as catalysis.^2,5^

Titanium-based MOFs are of particular interest due to the earth abundance of their metal source, high stability, low toxicity, redox activity, and promising photochemical properties.^6^ The latter pave the way for attractive applications, such as in photocatalysis, because tetravalent titanium cations exhibit an empty d-shell constituting the conduction band minimum, which can generate long-lived charge carrier, resulting in high solar-to-chemical conversion efficiencies.^7,8^ However, the synthesis of carboxylate-based Ti^4+^-clusters is challenging due to the high reactivity (prone to fast olation and oxolation) and the hydrolytic instability of titanium precursors, impeding the crystallisation process.^9–11^ Ti^4+^ ions are highly susceptible to hydrolysis, which results in the rapid formation of various molecular titanium-oxo-clusters and, eventually, in the precipitation of TiO_2_. As a result, significantly fewer titanium-based MOFs have been reported in the literature compared to other MOFs based on transition metals such as Zn^2+^, Cu^2+^, and Zr^4+^.^9,12–14^ Useful synthesis strategies for enabling the formation of Ti-MOF single crystals have been introduced by the Martí-Gastaldo group, *i.e.*, by metal doping^15^ or using hydroxamate-based linkers.^16^ Another strategy was proposed by Li *et al.* using a chelating coordination modulation method.^17^

One of the most interesting features of reticular materials is the combination of robust and flexible entities in a collective structure, which is also referred to as soft porous crystals^18,19^ or flexible framework materials^20–23^ which react to external stimuli (*e.g.*, temperature, pressure, light) by reversibly changing the framework structure without bond-breaking.^18,19^ These materials can respond to incorporation and release of guest molecules with structural deformations of the framework, resulting in expansion or shrinkage of the pore space, also referred to as breathing behaviour. Since then, framework materials have been classified into three categories according to their softness:^18,19^ flexibility through synergistic effects of the metal nodes' coordination symmetry, the rotatable axis of organic linkers, and strong host–guest interactions.^22,24–27^

Férey and co-workers first reported on the breathing/swelling behaviour in MOFs in 2002.^28^ To date, the dynamic behaviour of titanium-based MOFs is rarely described in the literature, and only a few reports are published.^9,29–31^ In COK-69, the framework flexibility results from the dynamic conformational change of the (*cis*/*trans*)1,4-cyclohexanedicarboxylate linker upon solvent removal.^9^ Another example is the heterometallic (TiMn_2_) MUV-35, where the single-crystal transformation is controlled by linker conformation in open, intermediate, and closed states.^30^

In the following, we report the synthesis, activation procedure, and the dynamic behaviour of the Ti-based MOF NTU-9 (NTU = Nanyang Technological University) triggered by guest adsorption and desorption. We optimised the synthesis parameters and evaluated for reproducibility towards large single crystals and microcrystalline bulk powders. Furthermore, we discovered a new metastable form NTU-9-d (d stands for distorted), which exhibits a strong pore and unit cell distortion upon activation. We investigated the structural transitions by *in situ* powder X-ray diffraction (PXRD) and THz Raman spectroscopy. These investigations revealed the first dynamic breathing behaviour of a titanium-based metal–organic framework with a rigid linker upon solvent loss and exposure to humidity/air. Our work establishes a reproducible synthesis procedure and a detailed mechanism of the previously unknown framework dynamics.

## Results and discussion

### Synthesis, reproducibility and materials characterisation

**Fig. 1:**
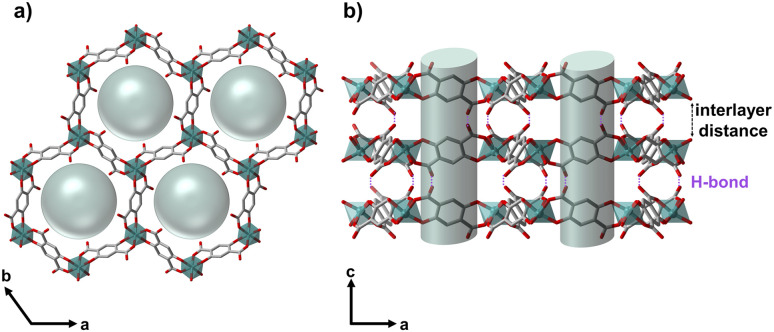
Image of the crystal structure of NTU-9 (ref. 32) (a) along the pore (view along *c*-axis) and (b) visualising the layer structure (view along *b*-axis), with the pores and pore content, *i.e.*, positionally disordered solvent molecules.

In 2014, Gao *et al.* synthesised NTU-9 (C_24_H_6_O_18_Ti_2_) as a photoactive titanium(iv)-based MOF with p-type semiconductor behaviour and utilised it for the photochemical degradation of organic dyes.^32^ This 2D MOF (Fig. 1) results from the reaction between titanium(iv)-isopropoxide and 2,5-dihydroxyterephtalic acid (H_4_dhta) in acetic acid, forming dark red hexagonal crystals composed of a single Ti ion-based hexagonal framework in an underlying hcb net.^32,33^ Later, investigations on NTU-9's efficient gas separation ability with an optimised synthetic procedure were reported.^34^

We first synthesised NTU-9 by reproducing different synthesis conditions reported in the literature.^32,35–37^ These synthetic routes differ in terms of solvent (acetic acid *vs. i*-PrOH : MeCN mixture) and reaction time (5 d *vs.* 1 d). Gao *et al.* reported the first solvothermal synthesis S1_5d_ (Solvent_reaction-time_) in acetic acid (1), acting as both modulator and solvent.^32^ The acidic medium during synthesis promotes a slow seeding by shifting the deprotonation equilibrium of the linker H_4_dhta toward the precursor side, thus slowing down the reaction, resulting in larger crystals.^33,38^ In contrast, Yu *et al.* used a 1 : 1 mixture of *i*-PrOH and MeCN (2) in an alternative synthesis S2_1d_ in the absence of modulator and decreased the reaction time to one day instead of five.^35^

As the limited reproducibility of MOF syntheses is a recurring problem that is gaining attention in the field,^39,40^ we statistically studied the influence of the reaction time for each synthetic protocol by performing the reaction between 4 and 21 times. Indeed, we found different trends depending on the solvent used (Table 1 and Fig. S1, S2†).

**Table 1:** Synthetic conditions for NTU-9 varying in solvent (acetic acid *vs. i*-PrOH : MeCN) and reaction time (between 15 min and 10 days). Reactions yielded an amorphous phase (red), phase mixture (yellow), or pure phase (green) of NTU-9. (successful synthesis/total reaction attempts)

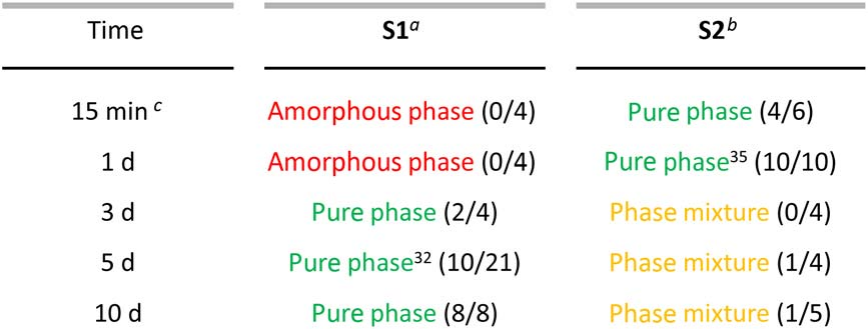

a Acetic acid as solvent.

b 1 : 1 ratio of*i*-PrOH : MeCN as solvent.

c Microwave-assisted reaction.

**Fig. 2:**
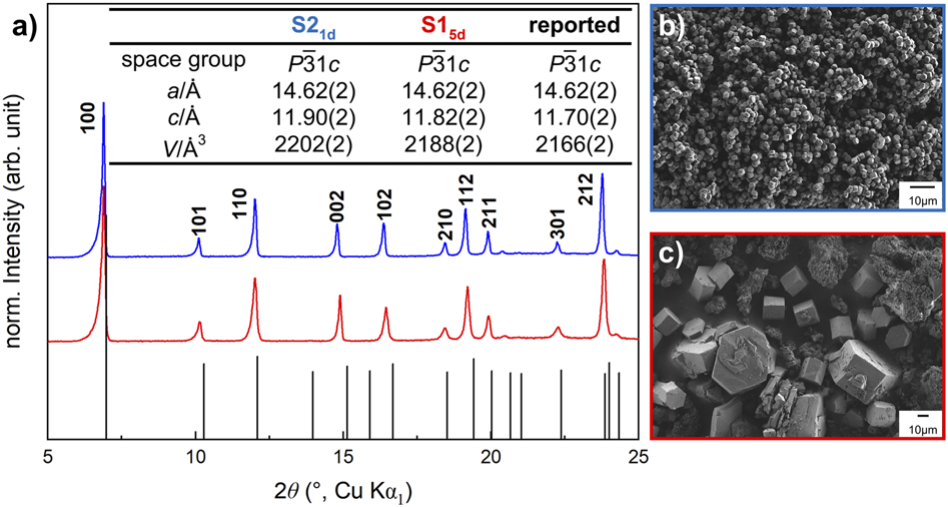
(a) PXRD pattern of NTU-9 calculated from the single crystal (black)^32^ including the refined and reported lattice parameters of the as-synthesised S1_5d_ (red) and S2_1d_ (blue) isolated crystallites. Inset: table with corresponding space group, refined lattice parameters, and unit cell volume. SEM images of (b) S2_1d_ and (c) S1_5d_.

Half of the S1_5d_ syntheses resulted in the formation of an amorphous product (Fig. S3†), as has been previously observed by other groups.^41^ The successful S1_5d_ attempts resulted in the formation of a heterogeneous mixture of dark red, crystalline hexagonal NTU-9 and microcrystalline powder (Fig. 2c and S4†). We confirmed the phase purity of the bulk powder by PXRD measurements (Fig. 2a). Scanning electron microscopy (SEM) revealed the formation of crystals with varying size (20–50 μm) and microcrystalline powder with average crystallite size smaller 0.5 μm (Fig. 2c). S2_1d_ syntheses also resulted in the expected phase and showed uniformly sized (≈2.5 μm) hexagonal red crystals (Fig. 2a, b and S4†). The refined lattice parameters (Fig. 2a, inset) differ slightly from the reported values for the crystallographic *c*-axis and, hence, cell volume.^32^

Additionally, FT-IR data^32,35,42^ (Fig. S5†) as well as THz Raman spectroscopy^43^ (Fig. S6†) of the synthesised solids align with the reported literature and indicate successful formation of NTU-9 for both synthesis pathways. More detailed information regarding the synthetic conditions can be found in the ESI.†

We were able to solve the reproducibility problem for S1 by doubling the reaction time (S1_10d_). SEM images of S1_10d_ revealed a uniform distribution of larger agglomerated hexagonal crystals (120 μm). Conversely, decreasing the reaction time to three days did not yield any significant difference (S1_3d_) in terms of reproducibility, and decreasing it further did not produce any crystalline product. The attempt to change from solvothermal to microwave-assisted synthesis to further reduce the reaction time, as shown by Yan *et al.*^37^ with formic acid, lead to amorphous products for the acetic acid based reaction.

On the contrary, we found that a microwave-assisted synthesis could reduce the reaction time to 15 minutes for the synthesis without modulator (S2_mw_), yielding a mixture of polycrystalline powder and different-sized hexagonal crystals. Instead, increasing the reaction time resulted in a phase mixture (S2_3d_, S2_5d_, S2_10d_). Additional broad reflections in the diffraction patterns could not be assigned to one of the reported polymorphs of NTU-9 ^17,44^ (Fig. S7 and S2†).

S1_3d_, S1_5d_, S1_10d_ and S2_mw_, S2_1d_ resulted in phase-pure NTU-9 with slight peak shifts in the PXRD patterns. The peaks of the resulting products of synthesis route S2 are slightly shifted to lower 2*θ* values, indicating a larger unit cell volume, compared to S1, which is confirmed by Rietveld refinement^45^ for each sample (Fig. 2, S8 and Table S1†). All products crystallise in the trigonal space group *P*3̄1*c*; but the *c*-axis increases for the S2. This variance may result from different pore fillings and solvent interactions. Despite multiple washing steps of each sample with ethanol, it is possible that a complete solvent exchange did not occur, and the reaction solvent might exhibit a stronger binding interaction with the framework. This leads to an extension or shrinking of the interlayer distance (Fig. 1b), which indicates a tendency for NTU-9 to be flexible towards different solvents.

### Stability towards solvents – guest molecules for structural stabilisation

**Fig. 3:**
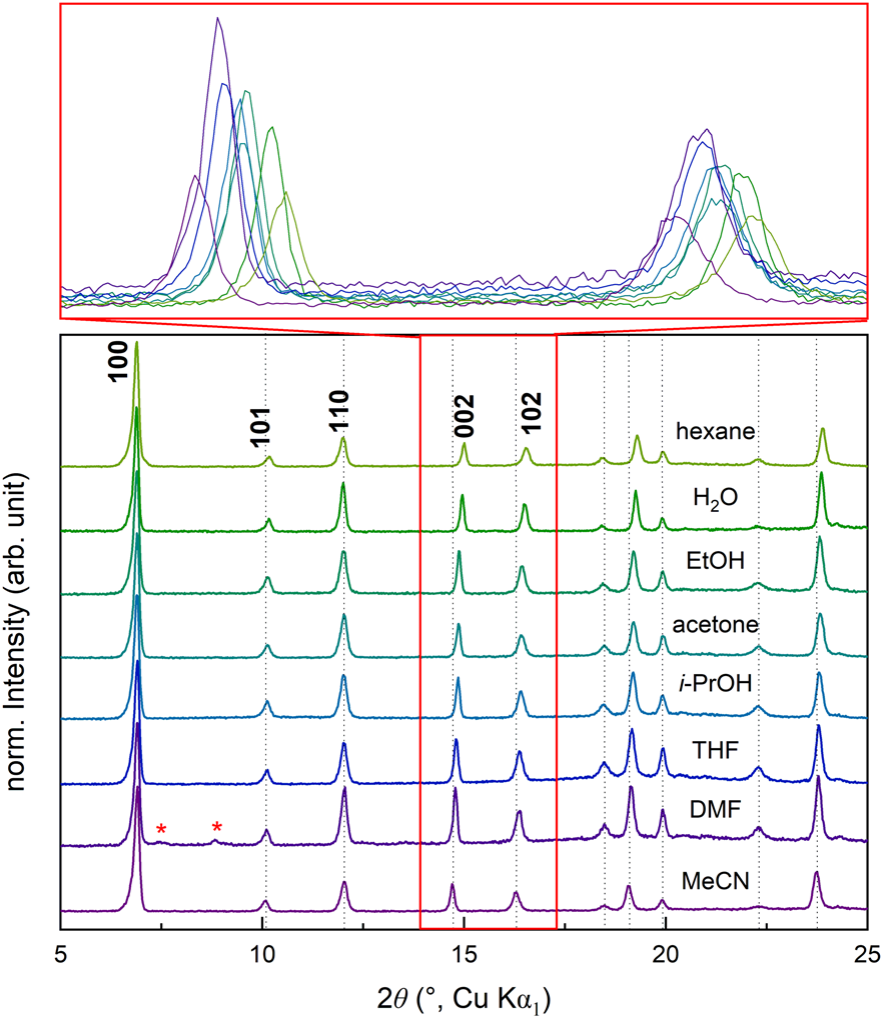
PXRD patterns of S1 with different solvents used for solvent-exchange. Dotted lines highlight the peak shifts and red asterisks indicate newly formed peaks.

We studied the framework–solvent interaction by solvent exchange of the incorporated solvent molecules, letting the NTU-9 crystals soak in polar and non-polar solvents (hexane, H_2_O, EtOH, acetone, *i*-PrOH, THF, DMF, MeCN) for three days at room temperature (Fig. 3). The overall PXRD patterns of the resulting solvent-exchanged NTU-9 samples can be indexed by the reported trigonal cell, which shows that the MOF is stable in different solvents. Some peaks, however, are significantly shifted, *e.g.*, 002 or 102. This could indicate that different-sized solvent molecules are accommodated within the pores. With different solvents incorporated, the unit cell volume slightly varies between 2174(2) Å^3^ and 2208(2) Å^3^. The volume change mainly originates from a slight change of the *c*-axis (Fig. S9 and Table S2†). This behaviour can be explained by the anisotropic structure of NTU-9 (Fig. 1): in the *ab*-plane, covalent and coordination bonds are stabilising the framework, which is chemically stiff. Perpendicular to the *ab*-plane, the 2D sheets are stabilised between each other by hydrogen bonds and van der Waals forces, which are chemically less strong and more elastic. The layered structure in the *c*-direction can thus be more easily expanded and accommodates different-sized solvent molecules, also depending on the specific solvent–framework interactions. After resuspension of NTU-9 in DMF, new, unindexed peaks emerge in the PXRD patterns, indicating the very slow formation of an additional, unknown phase (Fig. 3, S9 and S10†).

### Thermal stability at ambient pressure and under vacuum

**Fig. 4:**
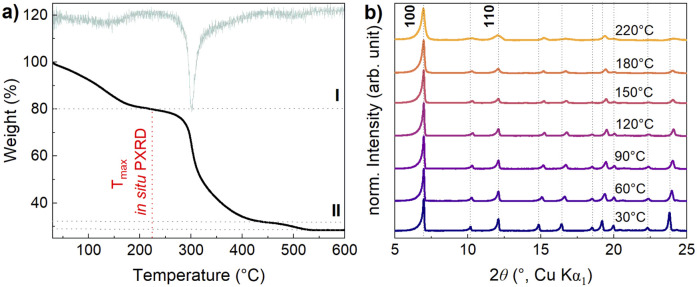
(a) TGA data of S1, heating rate 5 K min^−1^ under synthetic air atmosphere (flow rate 70 mL min^−1^) (black) and the derivation of the TGA data (light green). (b) Temperature dependent (30–220 °C) *in situ* PXRD measurements including selected reflection indices using an open capillary of S1. Dotted lines are included to indicate the peak shifts.

Next, we explore the thermal and solvolytic stability of NTU-9 (S1_5d_) in air and under dynamic vacuum. Thermogravimetric analysis (TGA) in synthetic air shows that the material is stable up to around 290 °C. Two main mass loss steps can be distinguished. Step I (20 wt% mass loss) in the temperature range of 30–220 °C can be assigned to the release of solvent molecules. The combustion of the linkers, which is associated with the deconstruction of the framework structure, leads to step II (48 wt% mass loss) in the range of 220–460 °C. At 460 °C, a small, additional mass loss occurs (3 wt% mass loss), and the TG-curve eventually reaches a plateau at 550 °C (Fig. 4a). *Ex situ* PXRD analyses show that combustion of NTU-9 leads to the formation of pure rutile (TG temperature 1000 °C). TGA was measured twice (5 K min^−1^*vs.* 10 K min^−1^) for the same batch to distinguish the solvent loss, which is assigned to be around 20 wt% (19 and 21 wt% for the different heating rates). We cannot be sure that all solvent is removed in the first step and remaining coordinating solvent molecules can be expected due to diffuse, residual electron density in the pores, as established by Rietveld refinement of the dried NTU-9 (Fig. S11–S13†).

For structural insights, we performed temperature-dependent *in situ* PXRD using the same sample (S1) within the temperature range of solvent release, *i.e.*, up to 220 °C. The heating results in changes in peak intensity, attributed to the release of adsorbed solvent molecules and peak broadening, indicating a decrease in crystallinity. The absence of a strong peak shift illustrates the low thermal expansion of NTU-9 and shows its rather strong thermal stability, as the overall pattern and, therefore, the framework of NTU-9 remains unchanged. The 100 and 110 peaks do not change positions but broaden, whereas the other peaks shift to slightly higher 2*θ* values, indicating smaller lattice parameters as the loss of guest molecules likely leads to a smaller lattice contraction (Fig. 4b).

**Fig. 5:**
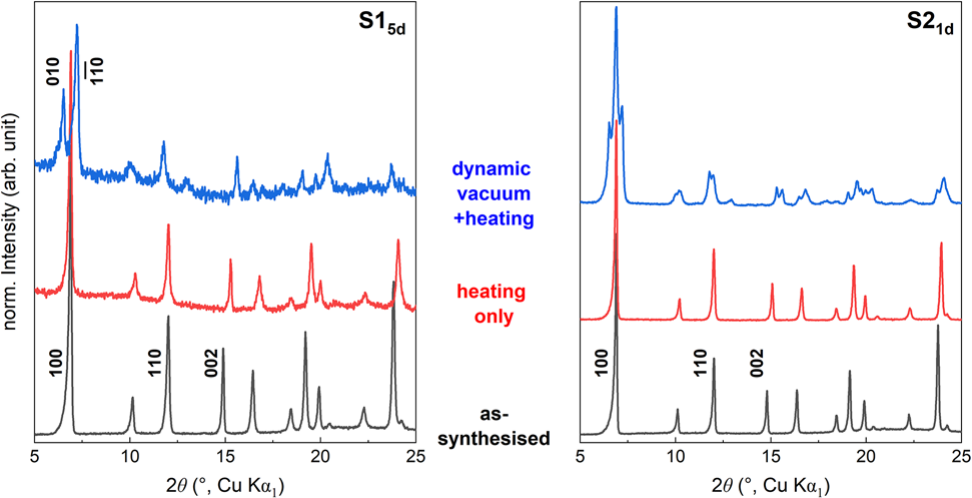
PXRD pattern of S1 (left) and S2 (right) at different stages of drying: as-synthesised (black), 120 °C heated (red) and exposed to dynamic vacuum at 60 °C (blue).

A typical activation procedure for MOFs employs heating under dynamic vacuum.^46^ Different activation procedures for NTU-9 are reported, *e.g.*, 120 °C under vacuum overnight,^32^ dried at 60 °C,^35^ without dynamic vacuum, or 80 °C under vacuum for 24 h.^36^ Surprisingly, PXRD revealed a different behaviour when activating NTU-9 using heat only (under ambient pressure) (Fig. 5, red lines) compared to applying additional dynamic vacuum (Fig. 5, blue lines). For the latter, we observed a pronounced peak splitting that implies a structural distortion. The distorted structure is characterised by a peak splitting of the 100 reflection and a shift of peaks, *i.e.*, 110 or 002, in the PXRD pattern. For S2_1d_, the conversion to NTU-9-d is incomplete, resulting in a phase mixture of NTU-9 and NTU-9-d. In the mechanistic study to understand the distortion, we focus on synthesis S1, which results in NTU-9-d upon evacuation and not in a phase mixture (Fig. 5, S14 and S15†).

### Characterisation of the new form (NTU-9-d)

**Fig. 6:**
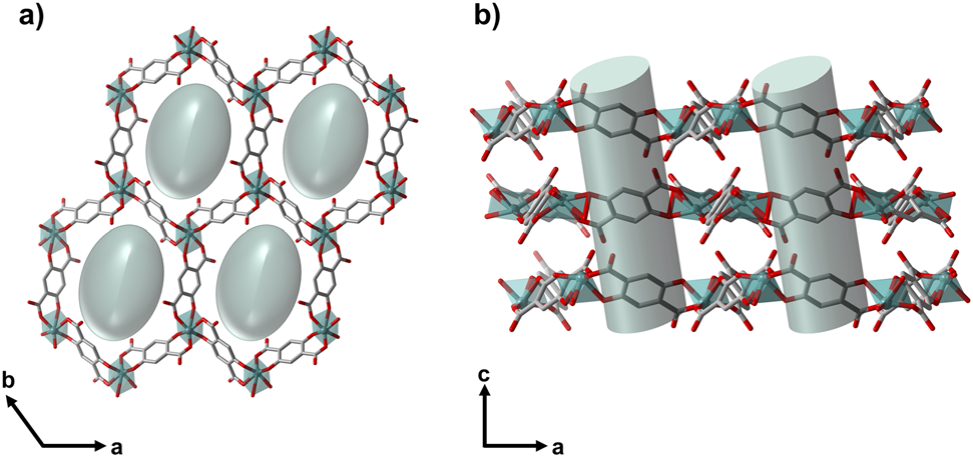
Structural model of NTU-9-d's distorted crystal structure upon vacuum exposure (a) in the *ab*-plane and (b) along the *c*-axis. Green ellipsoidal cylinders represents the random distribution of solvent molecules within the channels.

We derived a structural model of the distorted NTU-9-d from the PXRD data. Starting from the trigonal structure of non-distorted NTU-9, we gradually reduced the space group symmetry and performed global optimisation of the lattice parameters by performing multiple Rietveld refinements^45^ (further details are given in the ESI†). Eventually, we were able to describe the diffraction data of the distorted NTU-9 by using a triclinic unit cell with space group *P*1̄ (Table 2). After distortion, NTU-9 is contracted along the *a* axis and the *β* angle significantly decreases. This leads to a decrease of 9% in unit cell volume from 2192(2)Å^3^ to 1995(4)Å^3^ (activation *via* vacuum at 60 °C) and to an ellipsoidal pore distortion. (Table S3†) This behaviour is unusual in 2D frameworks as it is not the elastic axis *c* (layer distance) that changes, but the distortion occurs in the *ab*-plane, which results in the pore deformation. The pore channel running along the *c*-direction is slightly tilted and perpendicular to the *ab*-plane (Fig. 6). After the Rietveld refinement using only the node and the linkers, a significant amount of residual electron density is still visible within the pores (Fig. S13†). The broad, anisotropic peak shape of NTU-9-d indicates a significant structural disorder of the distorted framework. This led to instabilities in the final Rietveld refinements, when we attempted to refine the cation and linker position. Consequently, we can neither report reliable bond angles and lengths between the titanium nodes and the linker molecules nor quantify the residual amount of guest molecules within the pores.

**Table 2:** Cell parameters of NTU-9 and the resulting parameters of the model from NTU-9-d by different activation methods

Activation procedure	As-synthesised	120 °C, 7 h	120 °C, N_2_, 7 h	scCO_2_	Vacuum, rt	Vacuum, 60 °C
Polytype	NTU-9	NTU-9	NTU-9-d (83.8 wt%) NTU-9 (16.2 wt%)	NTU-9-d	NTU-9-d	NTU-9-d
Space group	*P*3̄1*c*	*P*3̄1*c*	*P*1̄	*P*1̄	*P*1̄	*P*1̄
*a/*Å	14.63(1)	14.62(1)	13.24(1)	14.15(1)	13.42(1)	13.24(2)
*b/*Å	14.63(1)	14.62(1)	15.23(1)	15.00(1)	15.36(1)	15.14(2)
*c/*Å	11.82(1)	11.53(1)	11.35(1)	11.50(1)	11.37(1)	11.31(2)
*α*/°	90	90	94.5(1)	92.3(1)	94.2(1)	94.3(1)
*β*/°	90	90	79.9(1)	85.6(1)	80.6(1)	79.9(1)
*γ*/°	120	120	116.0(1)	118.5(1)	117.1(1)	116.6(1)
*V/*Å^3^	2192(1)	2133(1)	2026(1)	2138(2)	2060(2)	1995(4)

We investigated the nature of this structural distortion by different activation methods: evaporation at room temperature/ambient pressure (overnight), heating at 120 °C under ambient pressure (7 h), heating at 120 °C under continuous nitrogen stream (7 h), supercritical CO_2_ activation (scCO_2_), and under dynamic vacuum (with or without additional heating). We observed that NTU-9-d formation was triggered by dynamic vacuum, scCO_2_, or heating in a continuous nitrogen flow, whereas drying the sample up to 220 °C at 1 bar (ambient pressure) did not distort the framework (Fig. 4b). The refined cell parameters of the differently activated samples S1 show that the cell distortion varies depending on the activation process. For the sample dried with only heat under nitrogen stream a mixture of NTU-9 and NTU-9-d is present. For the scCO_2_-activated sample, we observed less pronounced changes in the cell parameters compared to the vacuum-activated analogue; by dynamic vacuum exposure, we obtained a more distorted pore with an overall smaller cell volume, which can be amplified by additional heat. We can state that drying under vacuum distorts the pore most efficiently (Table 2, Fig. S16 and S17†).

Although vacuum-induced structural transformations in MOFs have been investigated,^47^ this is the first time that the flexibility behaviour of NTU-9 has been reported. We hypothesise that the differences between synthesis protocols (S1*vs.*S2) in the activation process (NTU-9-d *vs.* phase mixture) can be either explained by the difference in crystallite size which is known to impact dynamic framework transitions,^48^ or by the difference in the kind or position of the guest molecules within the pores. We noticed a difference by nuclear magnetic resonance (NMR) of the digested samples with and without modulator. In synthesis S1_5d_, the digested sample shows residual acetic acid (Fig. S18†).

To the best of our knowledge, no external stimuli-induced flexible behaviour for polymorphs of NTU-9 such as MIL-167-169 or FIR-117-119 has been reported as yet.^17,44^ However, some flexibility studies were reported for FIR-131-138, with sulfonated linker, when soaking crystals in different solvents.^49,50^

**Fig. 7:**
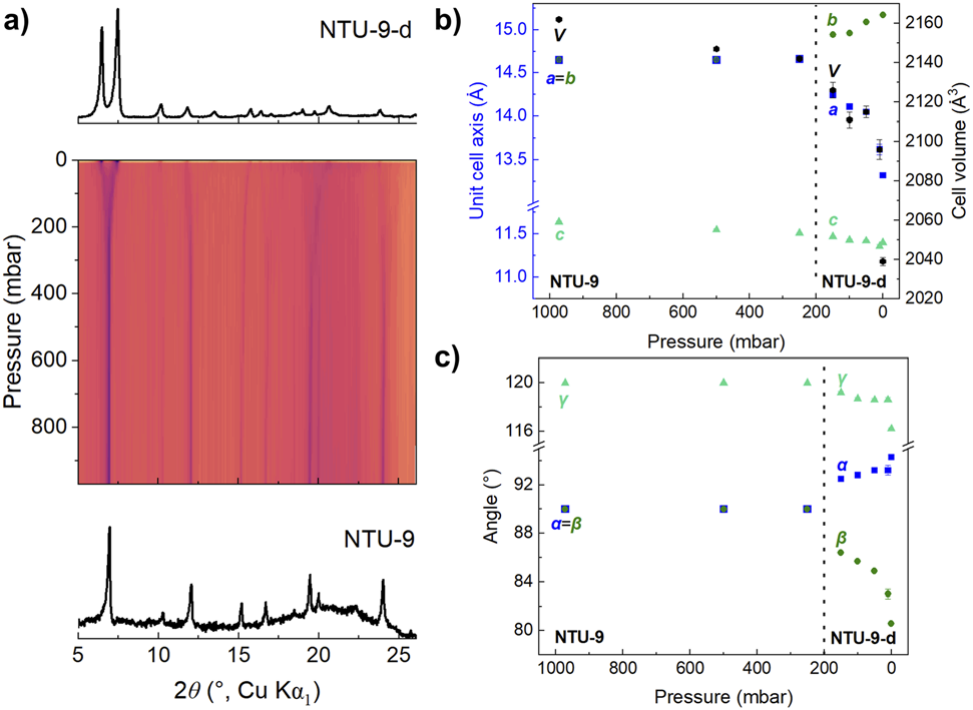
(a) *In situ* diffraction data of sample S1. The pressure was gradually reduced from ambient pressure to dynamic vacuum at 10 mbar at 60 °C. Evolution of (b) lattice parameter changes, volume changes, and (c) the unit cell angle changes during *in situ* vacuum PXRD.

To investigate the vacuum influence on the distortion of NTU-9 in more detail, we performed *in situ* PXRD under dynamic vacuum. Sample S1 was first heated to 60 °C followed by a stepwise reduction in pressure, eventually reaching a final dynamic vacuum of 3 × 10^−3^ mbar while constantly measuring diffraction patterns (Fig. 7a). At first, NTU-9 remained undistorted when heating at 60 °C and under ambient pressure. A slight decrease of the *c*-axis (Fig. 7b, green squares) indicates the incipient release of solvent molecules. Further pressure decrease to 200 mbar initiates the formation of NTU-9-d, indicated by the degeneration of the *a*- and *b*-axes and the *α*- and *β*-angles (Fig. 7b and c). The gradual distortion of the trigonal unit cell towards triclinic metrics and the reduction of the unit cell volume continues with further reducing the pressure. Interestingly, the transition from NTU-9 to its distorted form does not affect the contraction behaviour of the elastic *c*-axis.

The formation of NTU-9-d can also be followed by *in situ* THz Raman spectroscopy in dynamic vacuum at 60 °C. Changes from the initial spectrum compared to the vacuum evacuated sample (S1) at different pressure values between 0 and 1100 mbar and 60 °C can be observed (Fig. S6b and c†). The vibrations of the organic H_4_dhta linker dominate the Raman spectrum. Here we mainly focus on the Ti–O stretching band around 683 cm^−1^.^43,51^ The band broadening in the process of vacuum exposure indicates changes in the Ti–O bond upon pore distortion. We can assume that, as proven for Zr-MOFs, node distortion caused by guest removal/addition can also occur in Ti-MOFs.^52^ Broadening can also be observed in the symmetrical stretching vibration mode around 700 cm^−1^.^43^ This spectroscopic result hints at the formation of the distorted form NTU-9-d and, in particular, indicates changes in the octahedral titanium-linker coordination (Fig. S6b and c†).

CO_2_ gas adsorption experiments were conducted to analyse porosity and adsorption behaviour of NTU-9-d (S1). The CO_2_ adsorption isotherm was collected at 273 K and resembles a type I isotherm, indicating the present micro-pores (<2 nm) (Fig. S19a†). NTU-9-d adsorbed 72 cm^3^ g^−1^ of CO_2_ with a total pore volume of 0.15 cm^3^ g^−1^ and a calculated Brunauer–Emmett–Teller (BET) surface area of *S*_BET_ = 199 m^2^ g^−1^ (Fig. S19b†). The calculated pore size distributions is centred at 0.6 nm (Fig. S19c†). In comparison to a reported similar procedure for S2 from Cai *et al.* (2 d reaction time, without ethanol solvent exchange), the CO_2_ uptake of NTU-9 (no peak splitting observed) is about 12% higher with a largely higher BET surface area (917 m^2^ g^−1^ from N_2_ sorption data).^34^ These differences could originate from the tendency of S1 to distort, which causes lower accessibility of the material's pores, resulting in lower uptake and BET surface area. PXRD data after gas adsorption experiments indicate the stability of the distorted pore (Fig. S19d, S20 and Table S4†).

### Reversibility of the deformation of NTU-9

**Fig. 8:**
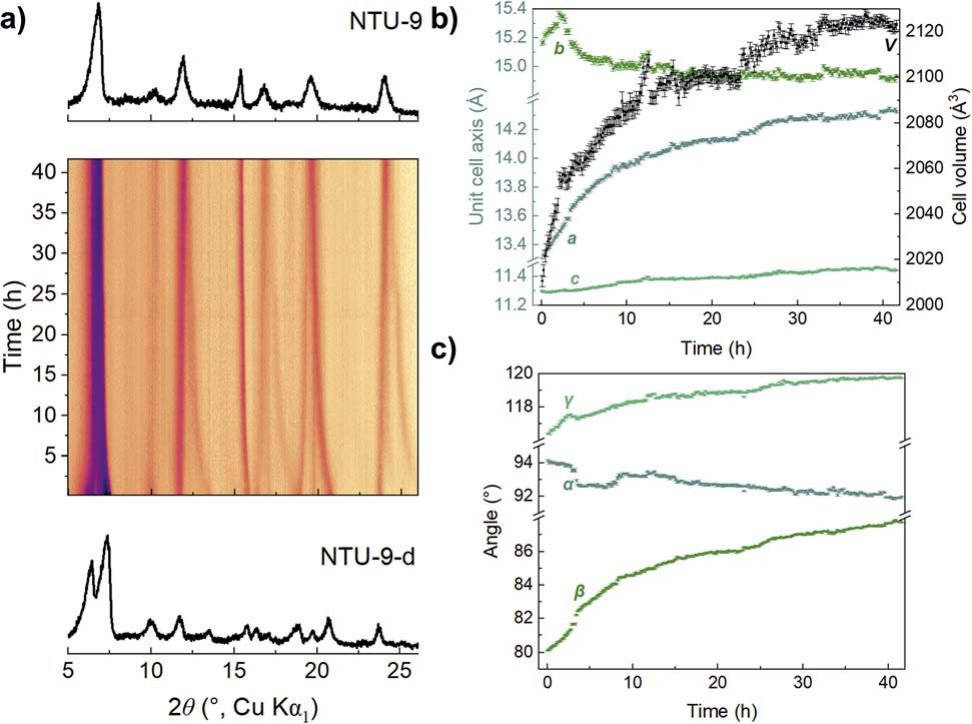
(a) *In situ* PXRD measurement of open capillary of vacuum dried S1 over time (RH of 25 ± 5%). Evolution of (b) lattice parameter changes, volume changes, and (c) the unit cell angle changes during the rehydration of NTU-9-d by ambient atmosphere.

After applying vacuum, NTU-9-d can be transformed back into its original state by exposure to moisture. We monitored the structural changes upon water uptake of NTU-9-d from the ambient atmosphere (22 °C, 25, 5% R.H.) in an open glass capillary with an opening diameter of 0.7 mm by PXRD over a period of 42 hours (Fig. 8).

The *c*-lattice parameter is hardly influenced by the water uptake (Fig. 8b, light green), whereas the *a*- and *b*-axis exhibit significant changes. Initially, both the *a*- and the *b*-axis expand upon exposure to ambient conditions (Fig. 8d, cyan and olive), which leads to a rapid increase in the unit cell volume (Fig. 8b, black). After approximately three hours, the *b*-axis starts contracting while a keeps expanding, leading to an overall expansion of the unit cell volume. Both unit cell axes become more similar in length, and the *γ*-angle between *a* and *b* expands towards 120° (Fig. 8c, light green). While the *α-*angle slightly decreases towards 90°, the *β*-angle rapidly expands to 90° (Fig. 8c, cyan and olive). Consequently, water uptake from the atmosphere distorts the ellipsoidal pore back towards the round shape of NTU-9, and the tilted channel becomes more perpendicular to the *ab*-plane. Despite the fact that the cell relaxes towards the trigonal metric of NTU-9, it does not reach this state after 42 hours of exposure to the ambient atmosphere, indicating a relatively slow water uptake from the atmosphere (Fig. S6d†).

We confirmed by *ex situ* PXRD that resuspending NTU-9-d in different solvents (ethanol, water, acetonitrile) overnight results in the recovery of NTU-9 (Fig. S21 and S22†). Different solvent molecules inside the filled channels lead to slight differences in the lattice parameters, in particular in the length of the elastic *c*-axis, of the resulting undistorted NTU-9 (Table S5), demonstrating the adaptive nature of the framework towards different guest molecules. Interestingly, we observe that the reversibility, from NTU-9-d to NTU-9, is significantly faster in the liquid phase compared to the gas phase. We demonstrated full reversibility of this desolvation-solvation process over 5 cycles by PXRD (Fig. S23†).

## Conclusions

In this work, we explore the impact and reproducibility of the synthesis conditions on the formation of the layered Ti-MOF NTU-9. We observe that the selection of solvent, additive, and reaction time is crucial for a reliable, reproducible synthesis of the phase pure material. Using pure acetic acid as modulator and solvent at 120 °C leads to the reproducible formation of large NTU-9 crystals. When long reaction times of at least 10 days are applied, the presence of acetic acid in the framework can be detected, even after vigorous post-synthetic washing steps. Solvothermal synthesis in an *i*-PrOH : MeCN mixture without a modulator at 120 °C is faster (1 day) and can be further sped up using microwave-assisted synthesis (15 min), but yields smaller NTU-9 crystals.

Pure heat treatment of the solvated MOFs up to 220 °C and ambient pressure removes large amounts of the incorporated guest solvent molecules without changing the framework's structure. However, activating the material with heating under a nitrogen flow, scCO_2_, or vacuum results in a previously unknown, distorted structure, abbreviated as NTU-9-d. The structural distortion transforms the circular pore channels into an ellipsoidal shape and reduces the unit cell volume by 9%. The distortion mechanism is primarily governed by compression within the hard coordination plane (*ab*-plane, metal–ligand coordination connectivity), with only minor changes in the elastic axis *c* (layer stacking *via* hydrogen bonding). This result is particularly interesting, as it contradicts the prevailing assumption that the soft connectivity between layers predominantly drives flexibility in 2D layered frameworks. Raman spectroscopy indicates a distortion of the octahedral coordination geometry of the titanium cations. Interestingly, the synthesis conditions strongly affect the flexibility of the MOF structure, where samples synthesised without modulator yield smaller crystals and show a lower tendency for distortion. The dried and distorted MOF is able to take up water from the atmosphere and a variety of polar and non-polar solvent molecules from the liquid phase, leading to a reversible structural relaxation into the undistorted form of NTU-9 over several cycles. Incorporating different solvent molecules into the structure of NTU-9 leads to significant changes in the elastic *c*-axis lattice parameters, which correspond to the interlayer distance. Despite the observed large flexibility of the framework, the long-range order is still maintained after multiple cycles of de- and resolvation and the associated pore distortion and relaxation.

This work illustrates the often-overlooked dynamic properties of reticular materials in general and Ti-based MOFs in particular under solvent evacuation/resuspension. Our study thus suggests that thorough characterisation of reticular materials for each process is essential to deeply understand framework behaviour and its resulting applications.

## Author contributions

J. E. K. and S. B. conceived the idea of the project. J. E. K. synthesised and characterised the metal–organic framework. F. H. performed CO_2_ sorption measurements. K. Gj. measured the *in situ* Raman data, A. S. the *in situ* vacuum PXRD data. S. B. performed the *in situ* rehydration measurements and the Rietveld refinements. J. E. K. wrote the manuscript with contributions from all authors. All authors read and commented on the final manuscript.

## Conflicts of interest

There are no conflicts to declare.

## Supplementary Material

SC-OLF-D5SC02585K-s001

## Data Availability

The data supporting this article have been included as part of the ESI.† The authors have cited additional references within the ESI.†^53–59^.
