# Supplementary material for: Dynamic breathing behaviour of the titanium-based metal–organic framework NTU-9 upon adsorption of water and organic solvents
Source: Chem Sci. 2025 Jun 25;16(30):13646–54. doi: 10.1039/d5sc02585k (PMC12208214; doi:10.1039/d5sc02585k)
Supplement: SC-016-D5SC02585K-s001 [file SC-016-D5SC02585K-s001.pdf]

Supplementary Information (SI) for Chemical Science.
This journal is © The Royal Society of Chemistry 2025

Dynamic breathing behaviour of the titanium-based metal-organic framework NTU-9 upon adsorption of water and organic solvents

Julia E. Knapp,[ab] Borja Ortín-Rubio,[a] Fabian Heck,[ac] Kristina Gjorgjevikj,[ab] Anastasia Sleptsova,[a] Simon Krause,[a] Sebastian Bette,[*a] and Bettina V. Lotsch[*abc]

[a] Max Planck Institute for Solid State Research, Heisenbergstraße 1, Stuttgart, 70569, Germany
[b] Department of Chemistry, University of Stuttgart, Pfaffenwaldring 55, Stuttgart, 70569, Germany
[c] Department of Chemistry, University of Munich, Butenandtstraße 5-13, Munich, 81377, Germany

*s.bette@fkf.mpg.de, *b.lotsch@fkf.mpg.de

Contents

S1. Materials, methods and characterisation 2
S1.1. Materials 2
S1.2. Methods 2
Synthesis (S1) of NTU-9 2
Synthesis (S2) of NTU-9 2
Synthesis ($S2_{mw}$) of NTU-9 3
S1.3. Characterisation 3
S2. Synthesis and reproducibility of NTU-9 6
S.2.1. SEM 6
S.2.2. PXRD analysis of different synthetic pathways 7
S2.3. Reproducibility of NTU-9 8
S2.4. Optical microscopy 8
S2.5. FT-IR spectroscopy 9
S2.6. THz Raman spectroscopy and in situ rehydration PXRD 10
S.2.7. PXRD of reported polymorphs of NTU-9 11
S.2.8. Rietveld refinements of the successful NTU-9 synthesis 12
S.2.9. Stability of S1 towards different solvents 14
S2.10. Thermogravimetric Analysis 17
S3. Characterisation of NTU-9-d 18
S3.1. Powder X-Ray Diffraction + Refinement 18
S3.2. Digest NMR 23
S5. Sorption Analysis 24
S4. Flexibility study 26
S4.1. Resuspension 26
S4.2. Cycles NTU-9 →NTU-9-d 27
S6. References 28

S1. Materials, methods and characterisation

S1.1. Materials

All reagents were used without further purification. 2,5-Dihydroxytherephthalic acid (CAS 610-92-4, 98%) was obtained from TCI. Titanium(IV) isopropoxide ($Ti(O^iPr)_4$) (CAS 546-68-9, 98%), glacial acetic acid (99.7%), and DMF (99.8%) were obtained from thermo scientific. 2-Propanol (99.9%), ethanol (99.8%), and acetonitrile (99.5%) were obtained from Roth. Tetrahydrofuran (THF, 99.5%), n-hexane (96%), and sodium hydroxide (95%) were purchased from Merck Supelco. Acetone (99%) was obtained from VWR Chemicals. Deuterium oxide (99.9%) was purchased by Aldrich.

S1.2. Methods

Synthesis (S1) of NTU-9

NTU-9 was synthesised by a modified method according to a previous method reported elsewhere.[1] Glacial acetic acid (4mL) was added to 2,5-dihydroxytherephthalic acid (150 mg, 0.76 mmol) in a 23 mL Teflon-lined stainless-steel autoclave. The yellow suspension was stirred for about 1 h, then titanium(IV) isopropoxide (200 µL, 0.67 mmol) was quickly added while continuous stirring. The suspension turned red and was stirred for 1 h. The mixture was transferred in the autoclave and was heated at 120 °C for 5 days. The mixture was cooled to room temperature and washed with ethanol (8x4 mL), resulting in dark red crystals.

The procedure of the reaction time-dependent experiments (1 day-10 days) was the same, except the changed reaction time.

Synthesis (S2) of NTU-9

NTU-9 was synthesised by a modified method according to a previous method reported elsewhere.[2] A mixture of isopropanol (4mL) and acetonitrile (4mL) was added to 2,5-dihydroxytherephthalic acid (204 mg, 1.03 mmol) in a 23 mL Teflon-lined stainless-steel autoclave. The yellow suspension was stirred for about 1 h, then titanium(IV) isopropoxide (75 µL, 0.25 mmol) was quickly added while continuous stirring. The suspension turned red and was stirred for 1 h. The mixture was transferred into the autoclave and was heated at 120 °C for 1 days. The mixture was cooled to room temperature and washed with ethanol (3x4 mL), resulting in dark red crystals.

The procedure of the reaction time-dependent experiments (1 day-10 days) was the same, except the changed reaction time.

Synthesis ($S2_{mw}$) of NTU-9

NTU-9 was synthesised by a modified method according to a previous method reported by another literature.[3] A mixture of isopropanol (4mL) and acetonitrile (4mL) was added to 2,5-dihydroxytherephthalic acid (204 mg, 1.03 mmol) in a 20 mL microwave vial. The yellow suspension was stirred for about 1 h, briefly sonicated, and then titanium(IV) isopropoxide (200 µL, 0.67 mmol) was added quickly while continuous stirring. The suspension partially turned red and was stirred for 1 h, quickly sonicated, and then microwaved at 120 °C for 15 min. The mixture was cooled to room temperature and washed with ethanol (3x4 mL), resulting in a powder consisting of red polycrystalline material.

S1.3. Characterisation

Powder X-ray diffraction (PXRD) Powder X-ray diffraction was recorded on a Stoe Stadi P diffractometer with Cu Kα_1 (λ = 1.540596 Å) source monochromatised with Ge(111), in a Debye-Scherrer geometry at room temperature. The samples were measured in ø0.5 mm or ø0.7 mm glass capillaries, and measured with spinning.

PXRD patterns for *in situ* de- and resolvation were collected on a laboratory powder diffractometer in Debye-Scherrer geometry (Stadi P-diffractometer (Stoe), Cu-Kα_1 radiation from primary Ge(111)-Johann-type monochromator and a triple array of Mythen 1 K detectors (Dectris), using a home-built gas loading setup based on a well established design.[4] The samples were filled in 0.7 mm borosilicate glass capillaries (Hilgenberg glass no. 0140) which were attached to a rubber plug. The capillaries were left open from one side, placed on the holder and tightly fixed with the metal cap. The sample holder was connected to the vacuum pump through a 4-way, allowing to control pressure and atmosphere in the capillary. During the PXRD measurements, the capillaries were rotated in an alternative fashion. Heating and cooling of the capillaries was realised by using a hot and cool air blower (Cobra 700, Oxford cryosystems).

PXRD data refinement The program TOPAS 6.0[5] was used for PXRD data analyses. The instrumental profile was described by the fundamental parameter approach implemented into the TOPAS software[6] and the precise lattice parameters were determined by LeBail fits[7] prior to each crystal structure refinement. The collected PXRD patterns of activated NTU-9 were subjected to

fully weighted Rietveld[8] refinements using the crystal structure model by Gao *et al.*[1]. Residual electron density in the pores caused by host molecules was modelled by using spherical dummy atoms (3-4 oxygen atoms per pore) with large thermal displacement parameters, in order to account for the positional disorder. The global optimisation method of simulated annealing[9] was employed to localise the molecules in real space, while the position of the titanium cations and the linkers was kept fixed. A structural model of NTU-9-d was derived by reducing the space group symmetry of NTU-9 to $P\bar{1}$. The global optimisation method of annealing was employed for optimisation of the lattice parameters, while the atom positions of the nodes and the linkers were kept fixed.

Optical microscopy An optical microscope Olympus BX51 was used. Images were recorded with the Olympus Stream Essentials 1.7 software.

Scanning electron microscopy (SEM) SEM SE (secondary electron) detector images were obtained on a Zeiss Merlin SEM microscope under the electron high tension voltage of 1.5 kV. The samples were placed in a carbon tape.

Infrared spectroscopy (IR) IR spectra were recorded on a Perkin Elmer UATR Two FT-IR spectrometer equipped with an attenuated total reflection (ATR) measuring unit.

Raman spectroscopy Raman spectra were recorded on a home-build system with a 785 nm laser equipped with a Raman Probe (Coherent TR-Probe 300 mW power at sample port, a steerable non-contact optic and a flood light module as accessories), connected to a spectrograph (Andor Kymera 328i, Andor iDUS 420 CCD detector). Measurements were performed at 30% laser power on the sample. The ambient temperature was recorded on samples in the same capillaries used for PXRD. For *in situ* measurements, the polycrystalline powder was transferred to a specific vacuum chamber within the Linkam THMS350V vacuum stage coupled with a T96-S temperature controller with the NEXUS software for computer control. The temperature was held at 60 °C and the pressure was varied with an oil free HiScroll 12 scroll pump from Pfeiffer.

Thermogravimetric analysis (TGA) TGA was performed on a NETZSCH STA 449 F3 Jupiter. Measurements were carried out with around 5 mg of sample in an Al_2O_3 crucible under synthetic air flow (70 mL/min) and a temperature range between 30 and 1200 °C and a heating rate of 10 K/min. Deviating buoyancy effects between the reference crucible and the sample-loaded crucible were compensated by a correction of y-offsets. Baseline correction was achieved by subtracting reference measurements with an empty crucible.

Sorption measurements CO_2 adsorption isotherms were recorded on a QUANTACHROME INSTRUMENTS Autosorb iQ MP at 273 K. The samples were degassed for 60 h at 6 °C under vacuum prior to the gas adsorption studies, if not stated otherwise. BET analyses were conducted using a program written by Alexander M. Pütz (https://github.com/AlexanderPuetz) based on the software BETSI by Osterrieth and Fairen-Jimenez.[10] The fitting ranges were selected to meet all four Rouquerol's criteria and to minimize the pressure error of monolayer loading. Pore size distribution was determined from CO_2 adsorption isotherms using the non-localized density functional theory (NLDFT) in carbon model for CO_2 at 273 K.

Nuclear magnetic resonance spectroscopy Liquid-state digest ^{1}H-NMR spectra were acquired using a JEOL ECZ 400S 400 MHz spectrometer. The residual solvent peaks serving as the internal reference. Approximately 4 mg of the sample were digested in 600 µL 1 M NaOH/D_2O.

Supercritical CO_2 activation was performed on a Leica EM CPD300 critical point dryer.

UV-vis Diffuse reflectance UV and visible light (UV-Vis) spectra were collected on a Cary 5000 spectrometer (referenced to barium sulfate) and the spectra were recorded in percentage of reflectance.

S2. Synthesis and reproducibility of NTU-9

S.2.1. SEM

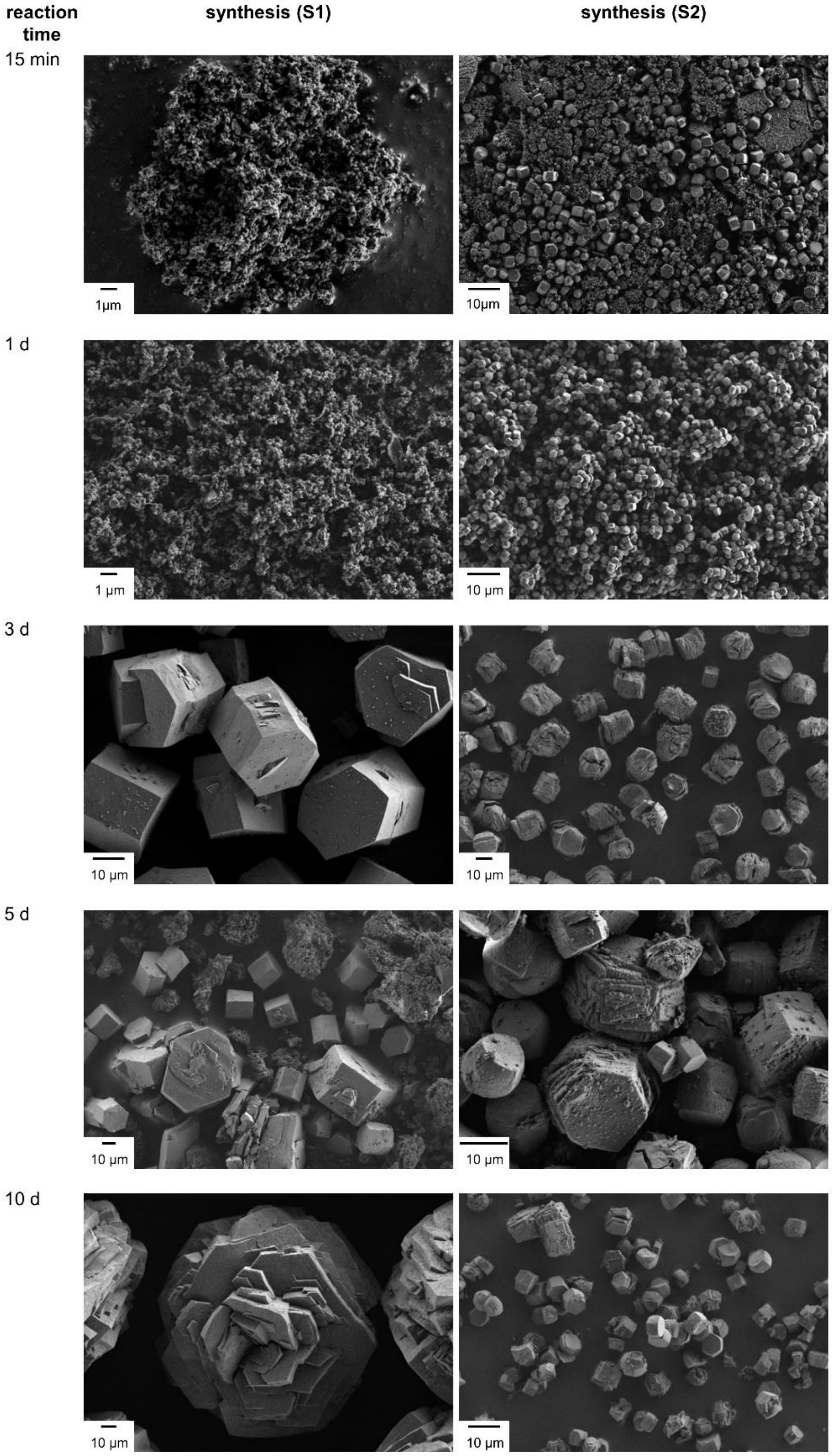

Figure S 1: SEM images of the products of synthesis in acetic acid, **S1**, and the synthesis without modulator, **S2**.

S.2.2. PXRD analysis of different synthetic pathways

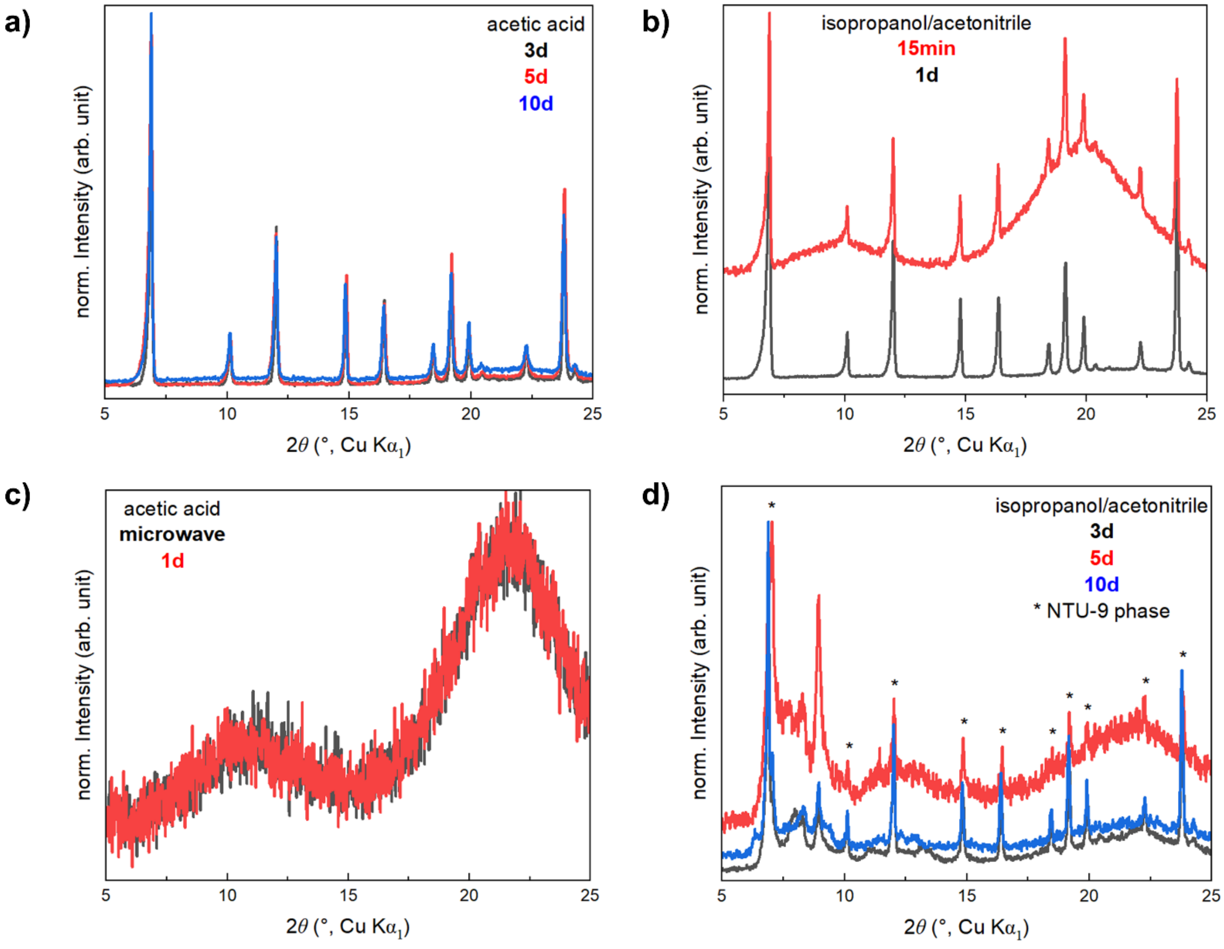

Figure S 2: Comparison of diffraction patterns of **S1** and **S2** with different reaction times. a) **S1**$_{3d,5d,10d}$ that yield in NTU-9. b) **S2**$_{mw,1d}$, yielding in NTU-9. c) **S1**$_{mw,1d}$, which did not yield NTU-9, but instead an amorphous product. d) **S2**$_{3d,5d,10d}$, which did not yield pure NTU-9, but with crystallises together with an additional phase.

S2.3. Reproducibility of NTU-9

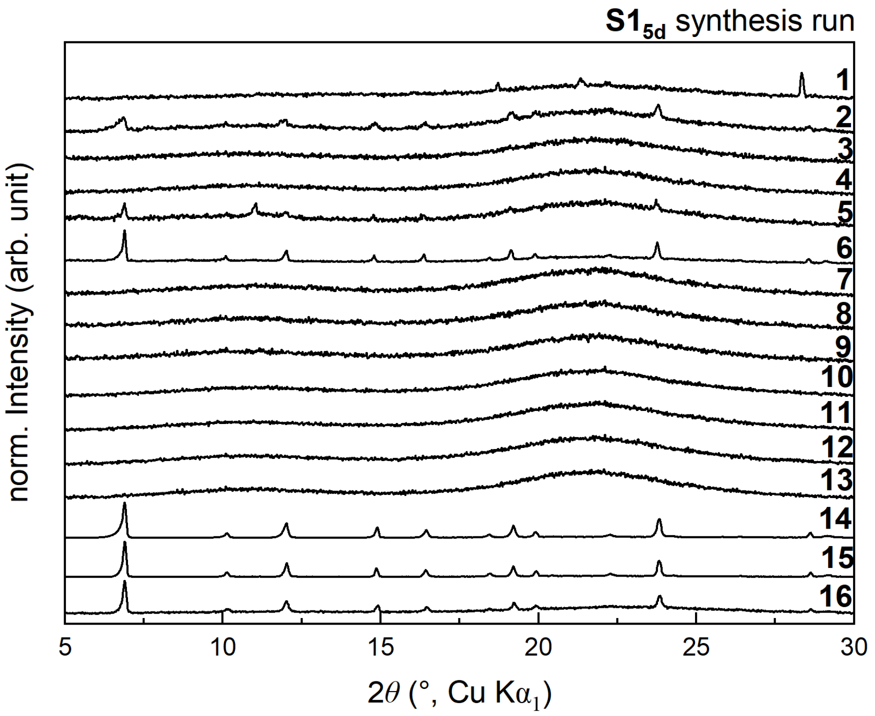

Figure S 3: Diffraction patterns of all **S1$_{5d}$** synthesis attempts (for synthetic run 14 five different batches and for synthetic run 15 two distinct batches were merged).

S2.4. Optical microscopy

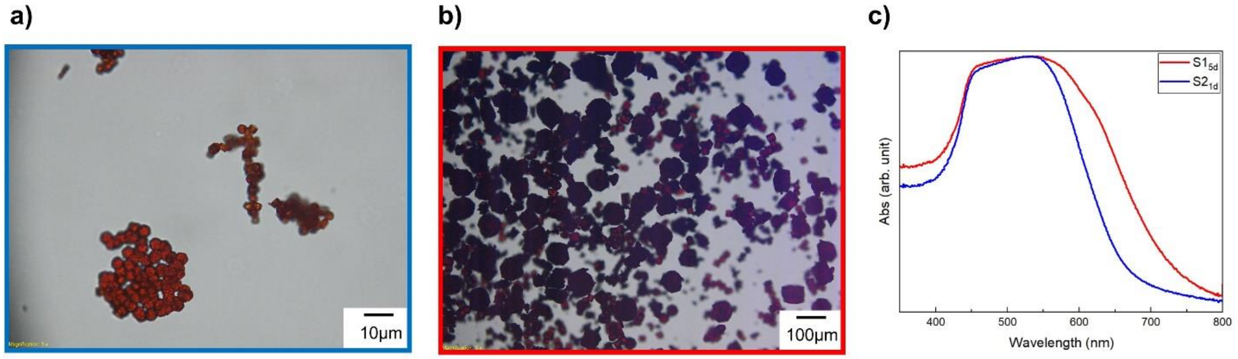

Figure S 4: Optical microscopy images of a) **S2$_{1d}$** and b) **S1$_{5d}$** and c) corresponding solid state UV-Vis data.

S2.5. FT-IR spectroscopy

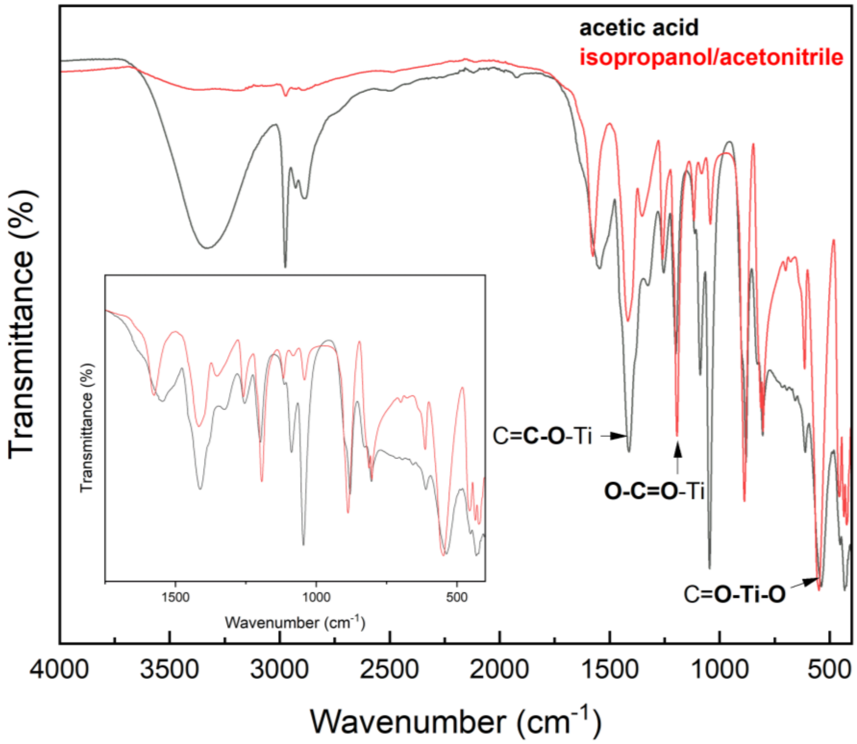

Figure S 5: FT-IR of as-synthesised NTU-9 of **S1**$_{5d}$ (black) and **S2**$_{1d}$ (red). The most characteristic bands, such as at 1190 cm^{-1} (**O-C=O**-Ti), 1410 cm^{-1} (C=**C-O**-Ti), and 535 cm^{-1} (C=**O-Ti-O**), are shared in both synthetic routes and confirm the Ti-coordinated MOF structure.[1, 2] However, some differences in peak intensity and peak position between **S1**$_{5d}$ and **S2**$_{1d}$ are present and might be explained by the slightly different cell parameters, solvent content, and the heterogeneous by-product. **S2**$_{5d}$ has a significant band around 3327cm^{-1} indicating the presence of hydroxyl groups, and might come from residual washing solvent.[11]

S2.6. THz Raman spectroscopy and in situ rehydration PXRD

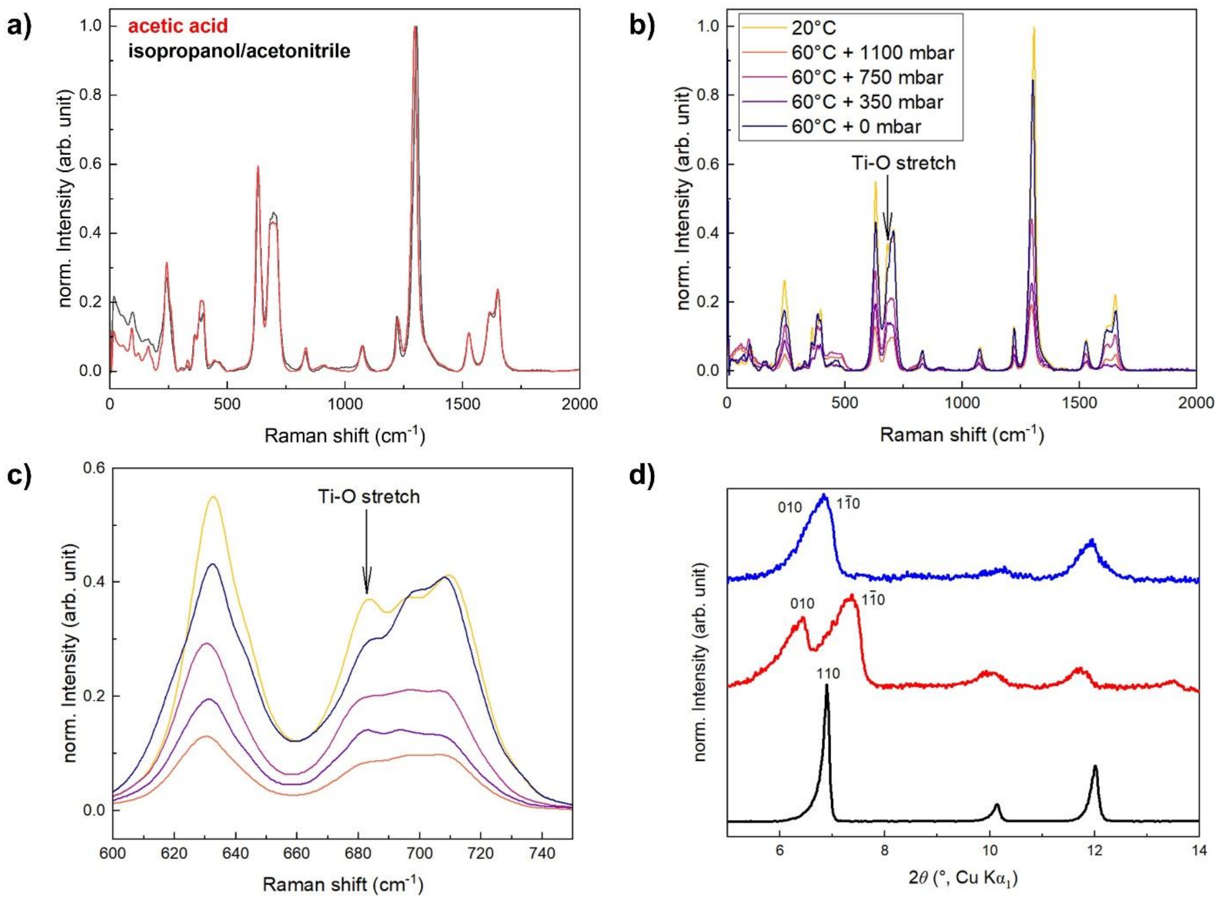

Figure S 6: a) Raman spectroscopy (excitation 785 nm) was measured on the capillaries used to record the diffraction data, **S2**$_{\mathbf{1d}}$ (black) and **S1**$_{\mathbf{5d}}$ (red). The vibration of the organic linker dominates the spectra. The Ti-O stretch is visible at 685 cm^{-1}, and the symmetrical stretching vibration is visualised by a band around 700 cm^{-1}.[12] b) *In situ* vacuum Raman data of **S1**. c) Close-up of the Ti-O-stretching in the Raman spectra. d) 100 diffraction peak of the as-synthesised NTU-9 **S1** (back), the vacuum dried NTU-9-d (red) and the rehydrated from after 42 hours exposure to ambient humidity (blue).

S.2.7. PXRD of reported polymorphs of NTU-9

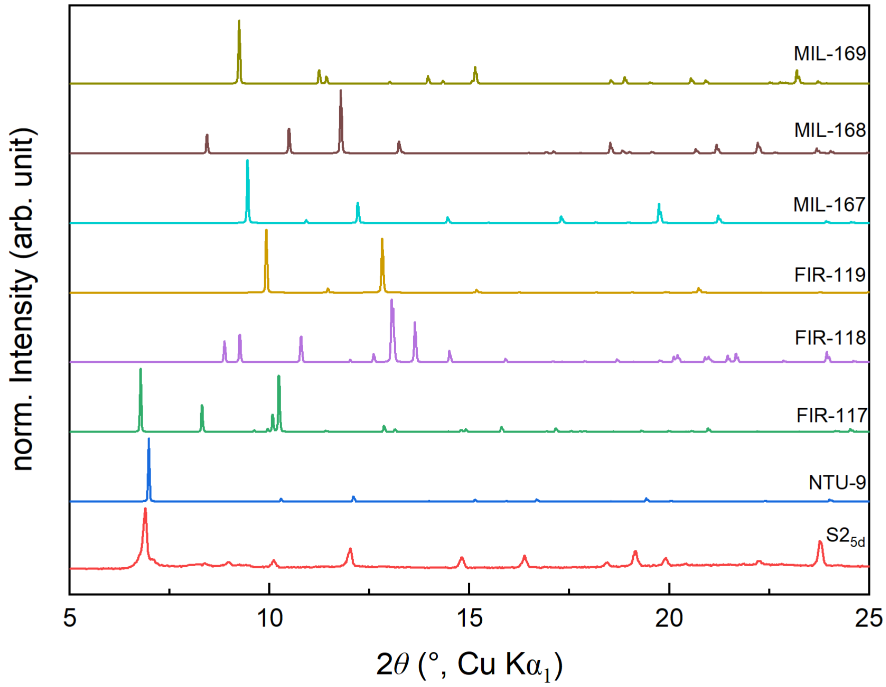

Figure S 7: Comparison of PXRD patterns of the Ti-MOFs (MIL169-167, FIR-119-117, NTU-9) with 2,5-dihydroxytherephthalic acid as linker, and the diffraction pattern of **S2$_{5d}$** with the non-identifiable additional phase.

S.2.8. Rietveld refinements of the successful NTU-9 synthesis

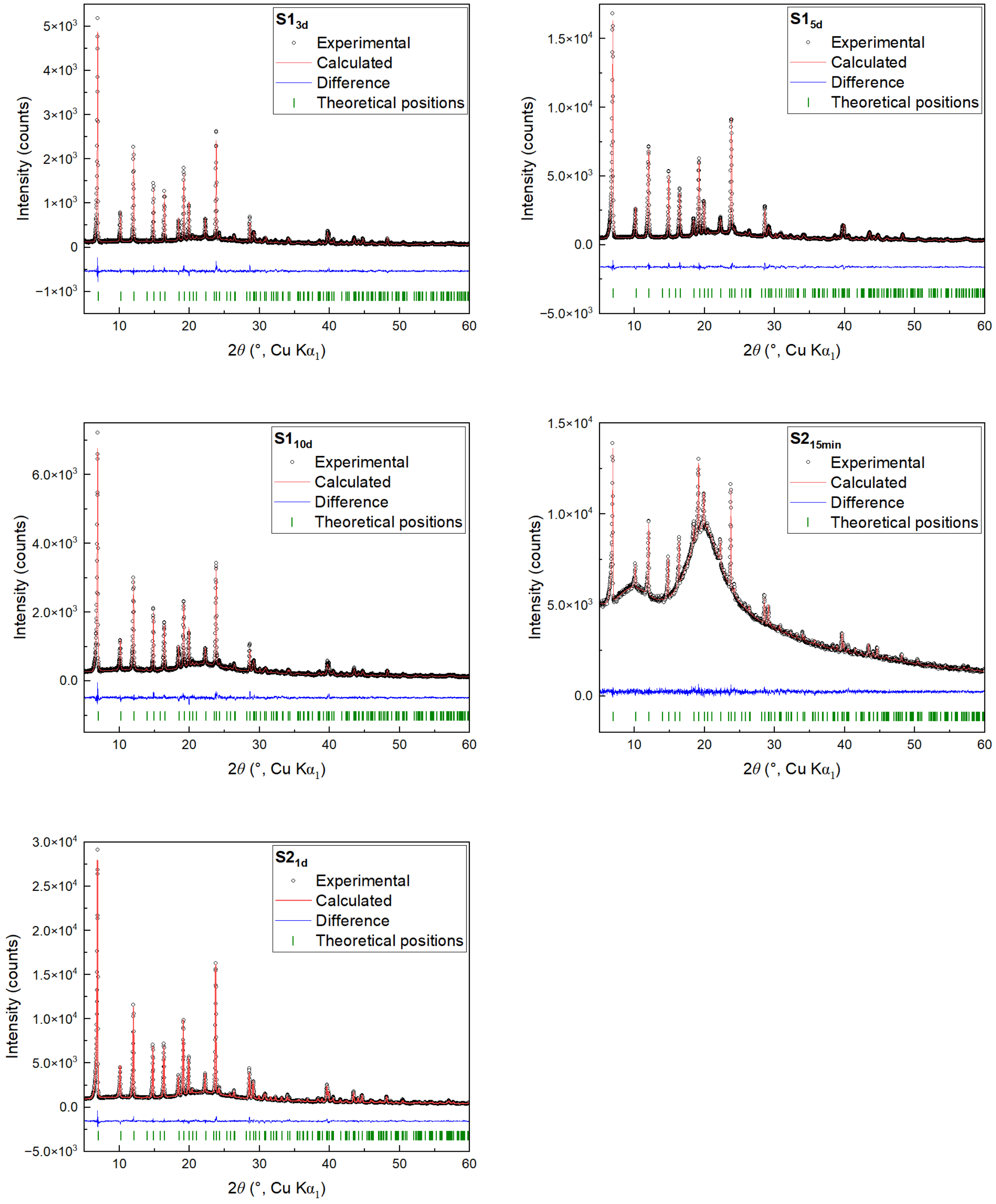

Figure S 8: Rietveld refinements of the successful NTU-9 synthesis **S1** and **S2** as a function of the reaction time.

Table S 1: Refined cell parameters, cell volume and space group of NTU-9 resulting from synthesis **S1** and **S2** applying different reaction times.

	$\mathbf{S1_{3d}}$	$\mathbf{S1_{5d}}$	$\mathbf{S1_{10d}}$	$\mathbf{S2_{15min}}$	$\mathbf{S2_{1d}}$
space group	$P\bar{3}1c$	$P\bar{3}1c$	$P\bar{3}1c$	$P\bar{3}1c$	$P\bar{3}1c$
a/Å	14.61(2)	14.62(2)	14.61(2)	14.62(2)	14.62(2)
c/Å	11.84(2)	11.82(2)	11.85(2)	11.90(2)	11.90(2)
V/Å^3	2189(2)	2188(2)	2191(2)	2205(2)	2202(2)

S.2.9. Stability of S1 towards different solvents

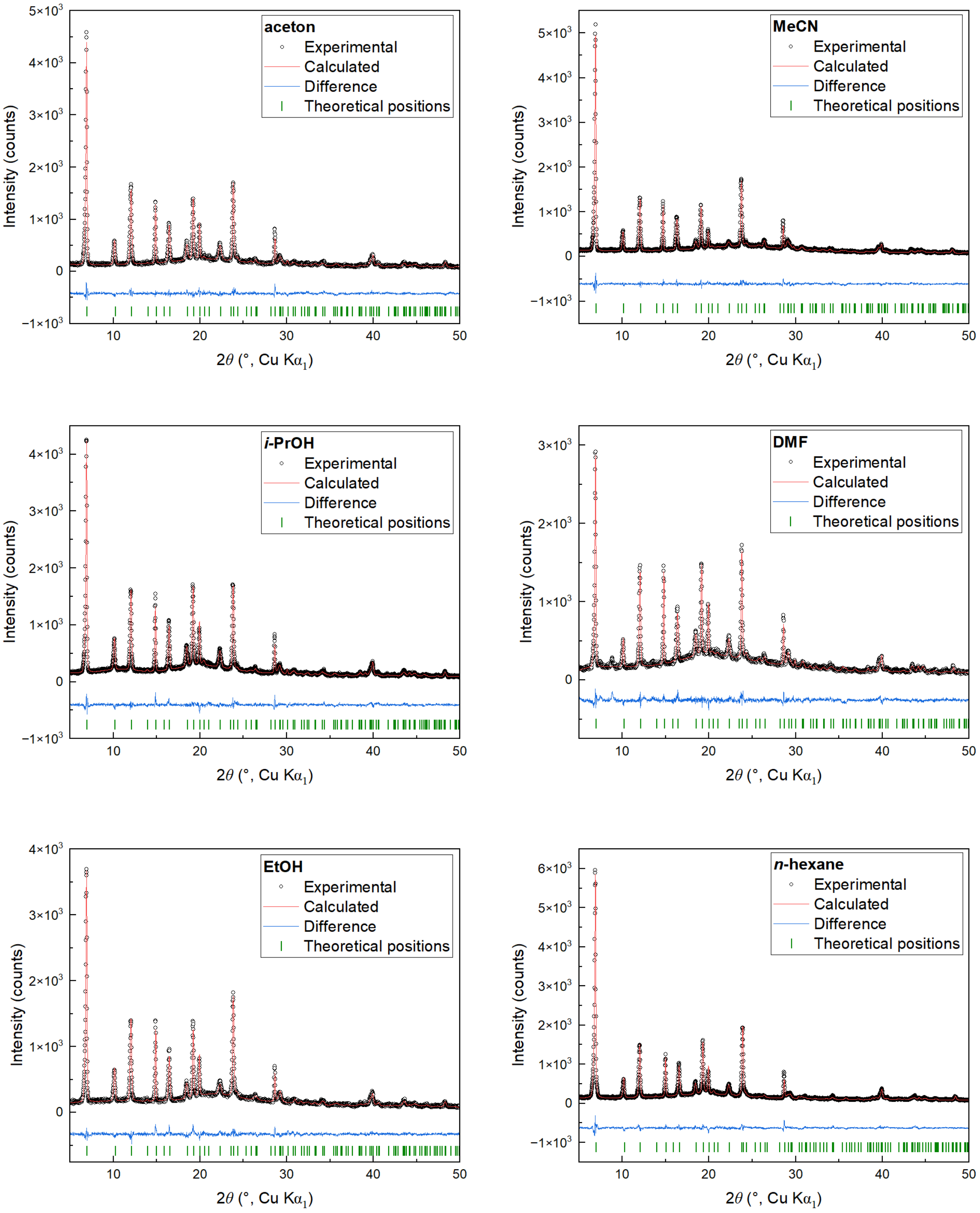

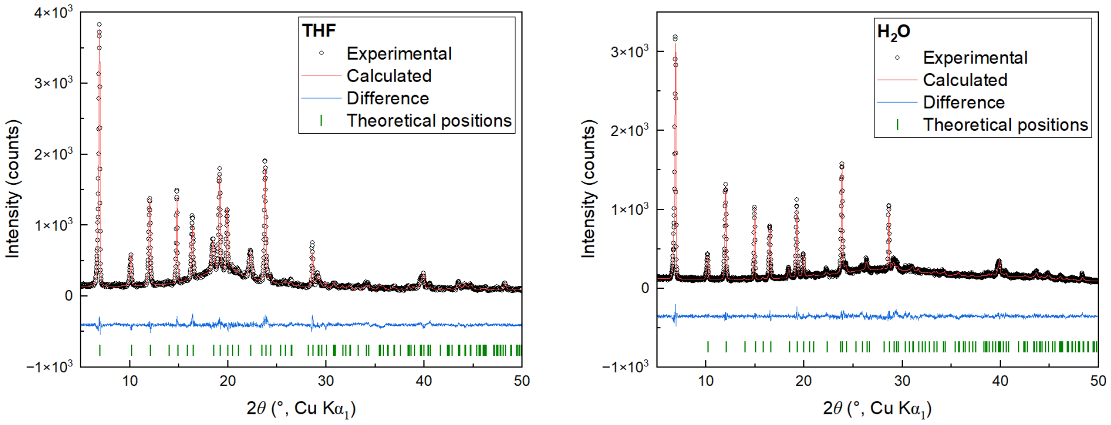

Figure S 9: Rietveld refinements of solvent exchanged **S1** in acetone, DMF, ethanol, hexane, 2-Propanol, acetonitrile, THF, and water.

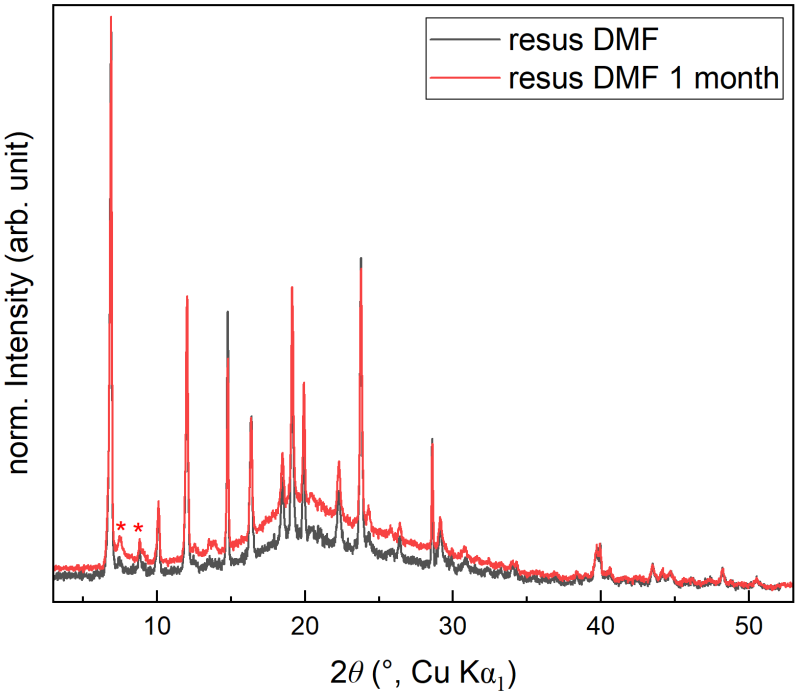

Figure S 10: PXRD patterns of **S1** resuspended in DMF for three days (black) vs. one month (red).

Table S 2: Rietveld refinement data with resulting space group $P\bar{3}1c$, cell parameters and cell volume of solvent exchanged **S1** in acetone, DMF, ethanol, hexane, 2-Propanol, acetonitrile, THF, and water.

	**EtOH**	**H_2O**	***i*-PrOH**	**acetone**	**MeCN**	**DMF**	**THF**	**hexane**	**NTU-9**
a/Å	14.61(2)	14.63(2)	14.61(2)	14.60(2)	14.60(2)	14.59(2)	14.60(2)	14.62(2)	14.62(2)
c/Å	11.83(2)	11.77(2)	11.86(2)	11.84(2)	11.97(2)	11.90(2)	11.89(2)	11.74(2)	11.70(2)
V/Å^3	2188(2)	2182(2)	2191(2)	2185(2)	2208(2)	2196(2)	2194(2)	2174(2)	2166(2)

S2.10. Thermogravimetric Analysis

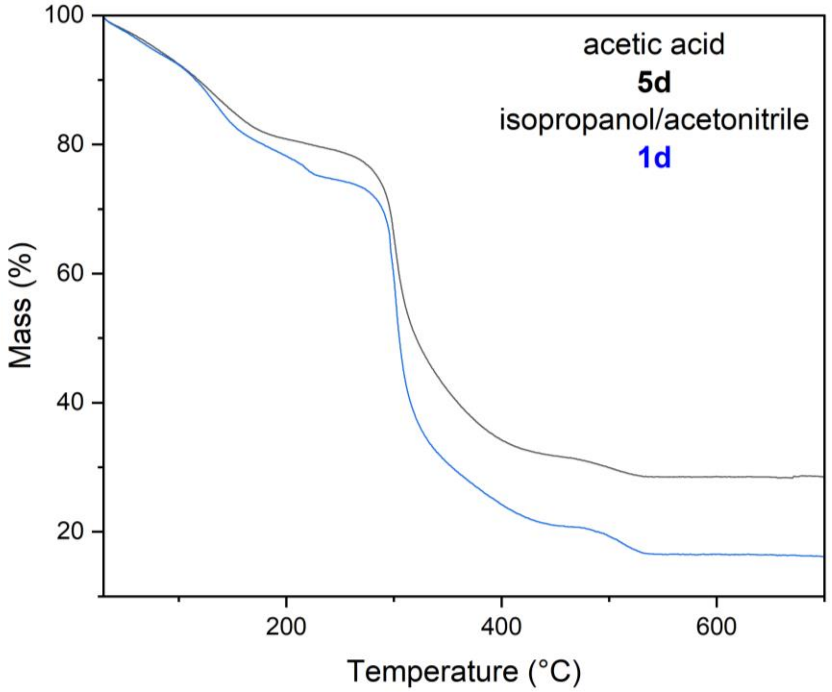

Figure S 11: TGA data of syntheses of **S1** and **S2** conducted with different reaction times. We see changes in the TGA curve (starting at around 120 °C) between **S1**$_{5d}$ and **S2**$_{1d}$. **S2**$_{1d}$ (16.5 wt%) has a higher mass loss at 600 °C than **S1**$_{5d}$ (28.5 wt%). Different pore contents might explain this behaviour. The difference in final mass loss correspond to different amounts of residual TiO_2 species.[13]

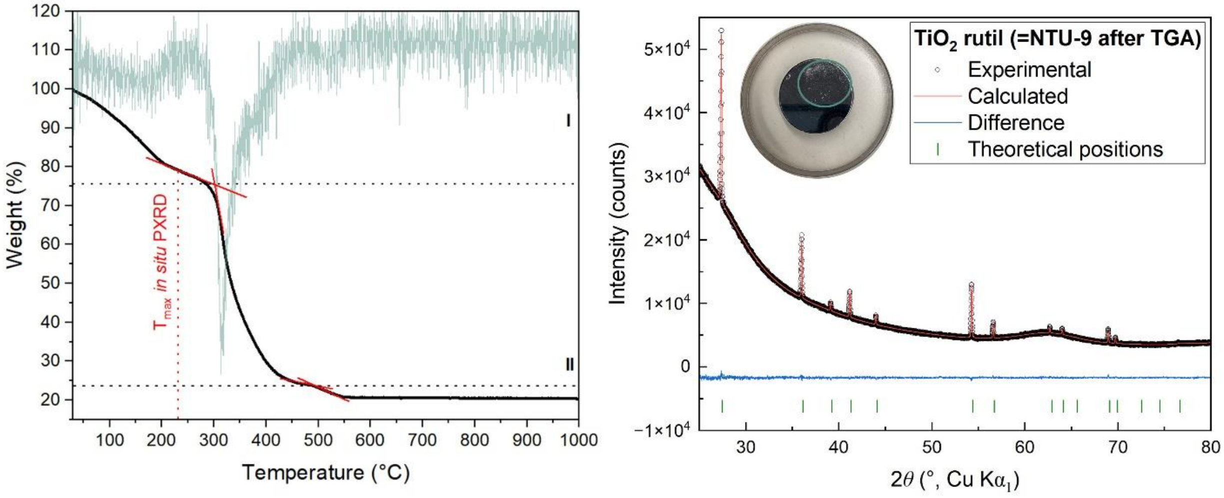

Figure S 12: TGA data of **S1** (left) and PXRD data of the resulting powder TiO_2 (rutile) after TGA, inset: flat plate with in green marked sample after TGA (right).

S3. Characterisation of NTU-9-d

S3.1. Powder X-Ray Diffraction + Refinement

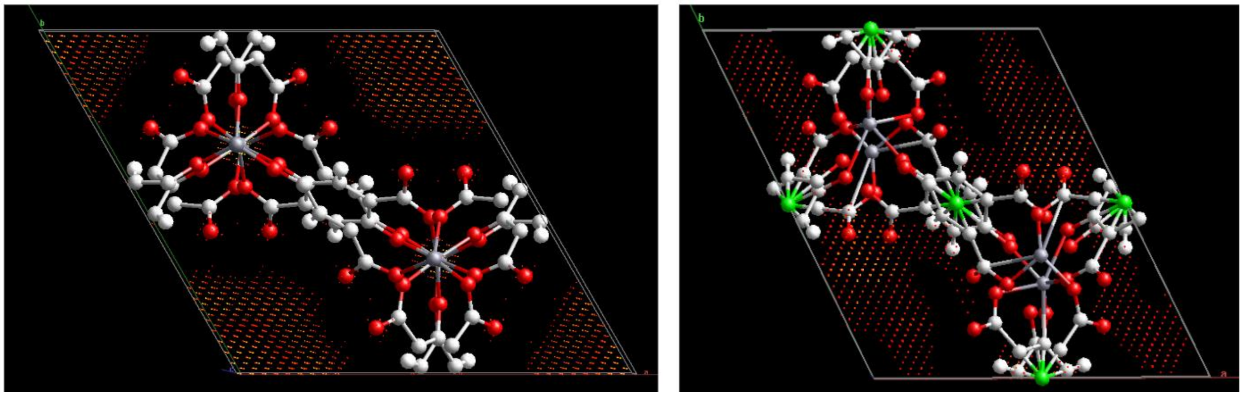

Figure S 13: Fourier map of NTU-9 (left) and NTU-9-d (right), the green atoms are dummy atoms with a site occupancy factor of 0 for positioning the rigid body describing the linker.

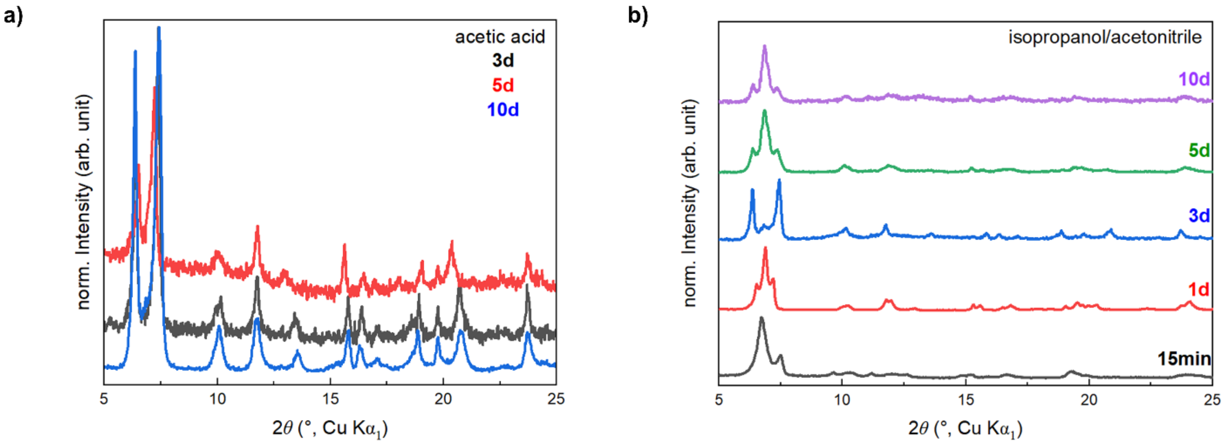

Figure S 14: Vacuum dried samples of a) **S1** and b) **S2** resulting in NTU-9-d or a phase mixture of NTU-9 and NTU-9-d.

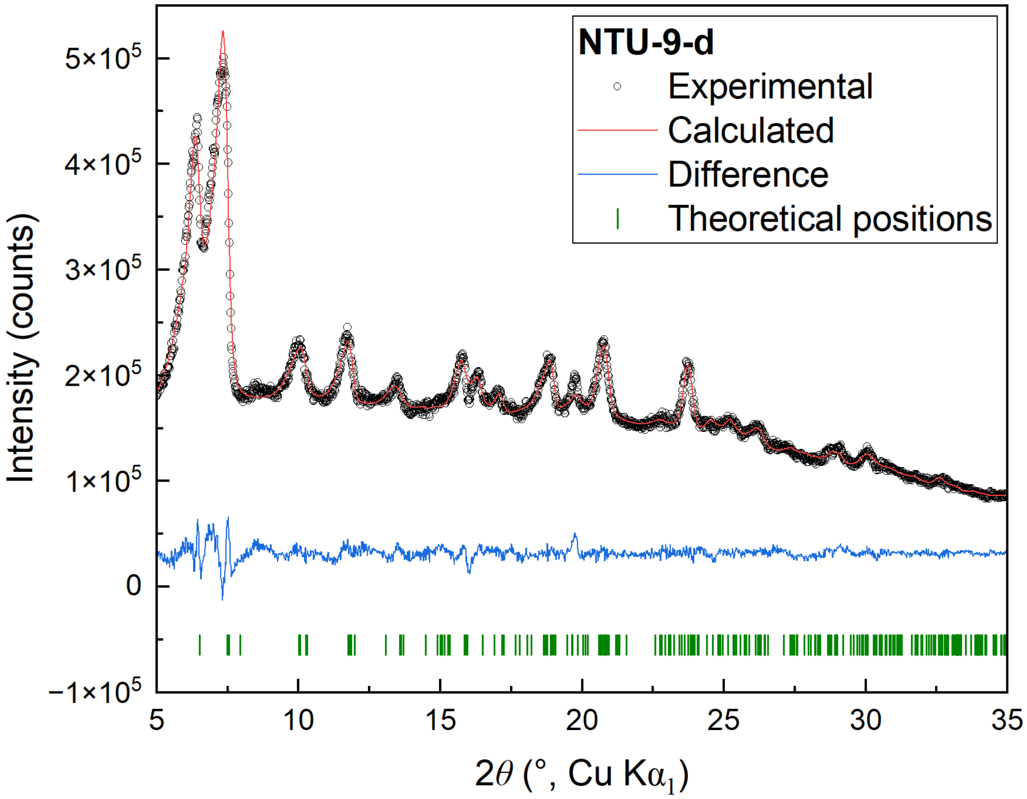

Figure S 15: Rietveld refinement of NTU-9-d with a R_{wp}=2.49%, R_p=1.95% and R-F^2=1.23%.

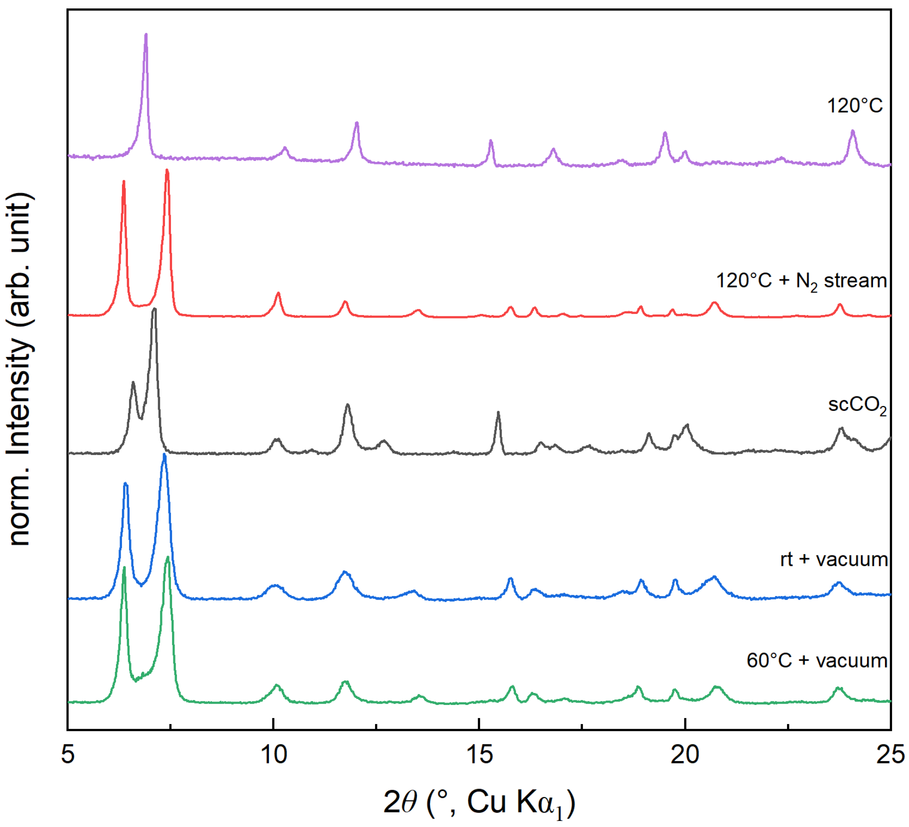

Figure S 16: PXRD patterns of a sample of **S1** dried under different conditions.

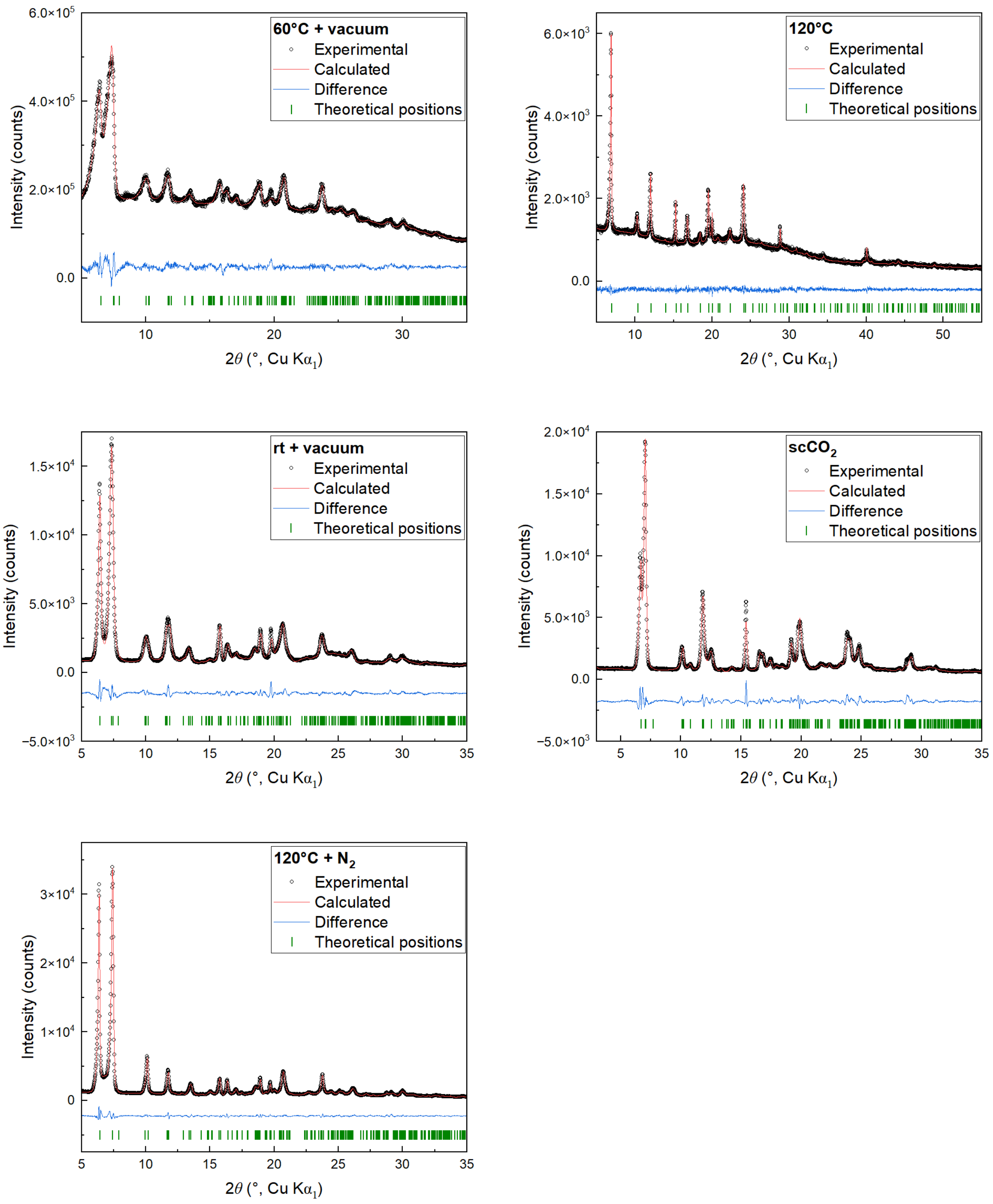

Figure S 17: Rietveld refinements of NTU-9-d activated **S1** with different procedures ($scCO_2$, vacuum at room temperature, vacuum at 60 °C).

Table S 3: Comparison of different observed cell volume changes in ´breathing´ MOFs.

		**Initial cell volume (Å³)**	**Contracted cell volume (Å³)**	**Percentage (%)**	**Reference**
Ti-based MOFs	NTU-9	2192(1) NTU-9	1995(4) NTU-9-d	9	this work
	MUV-35	15673.5(9) MUV-35-*o*	9630.9(5) MUV-35-*c*	39	[14]
	COK-69	2096.0(4) $COK\text{-}69_{op}$	1644.8(2) $COK\text{-}69_{cp}$	21	[15]
other metal-based MOFs	MIL-53	1383 MIL-53 *as* (Al)	706 MIL-53 *ht* (Al)	49	[16]
	MIL-88A	2110 (water)	1135	46	[17]
	MIL-88D	7968	2392	70	[18]

S3.2. Digest NMR

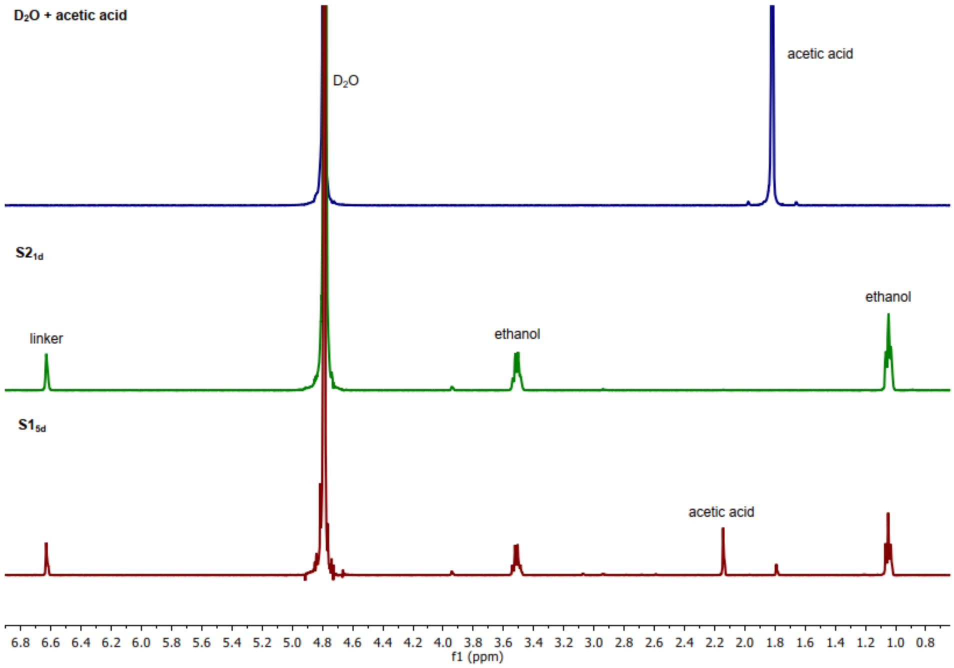

Figure S 18: ^{1}H NMR of digested **S2$_{1d}$** (green) and **S1$_{5d}$** (red) (400 MHz). Approximately 4 mg of the sample was digested in 600 µL of 1 M NaOH/D_2O. As comparison, 600 µL of 1 M NaOH/D_2O with additional 10 µL of acetic acid.

S5. Sorption Analysis

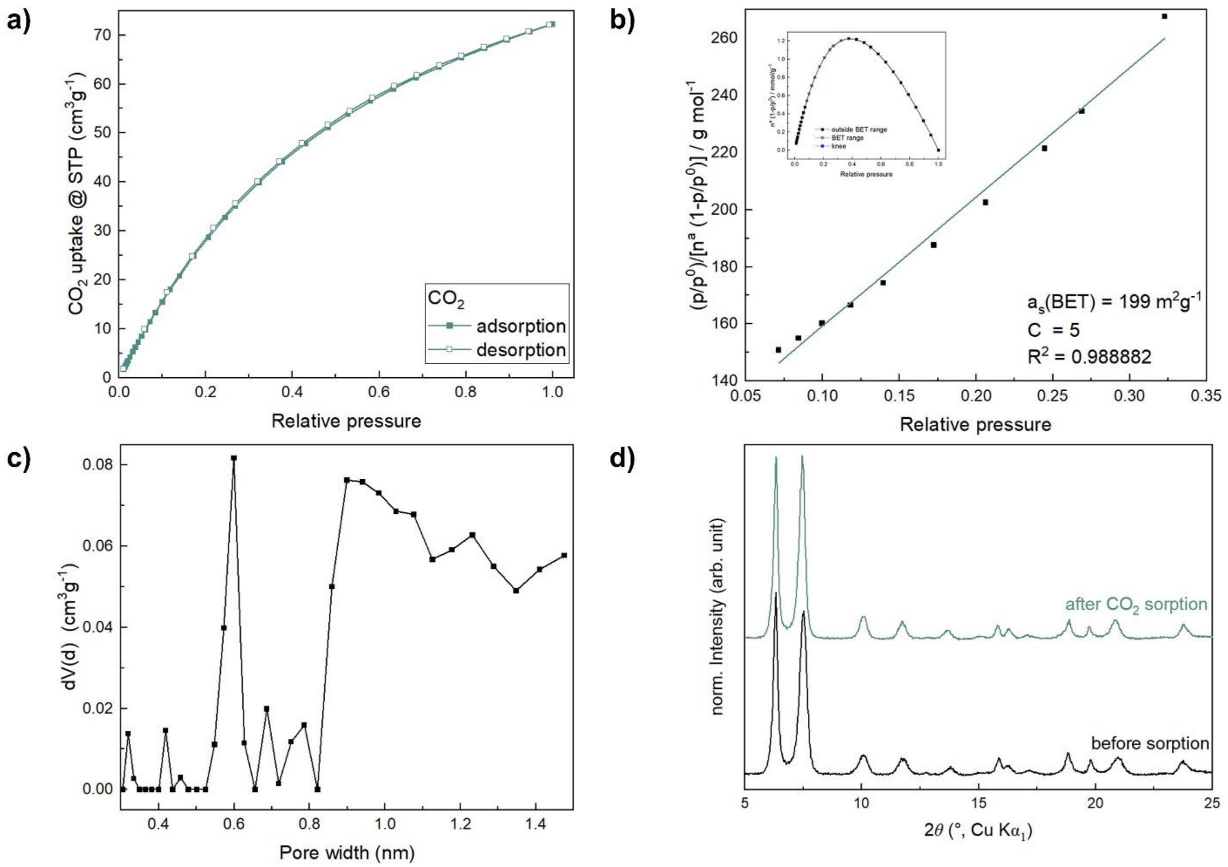

Figure S 19: a) CO_2 sorption isotherm of NTU-9-d at 273 K. **S1** was evacuated prior to the measurement at 60°C under dynamic vacuum (8 h). b) BET analysis. Inset: Roquerol representation. c) Pore size distribution. d) PXRD before (black) and after CO_2 (turquoise) sorption measurements of **S1**.

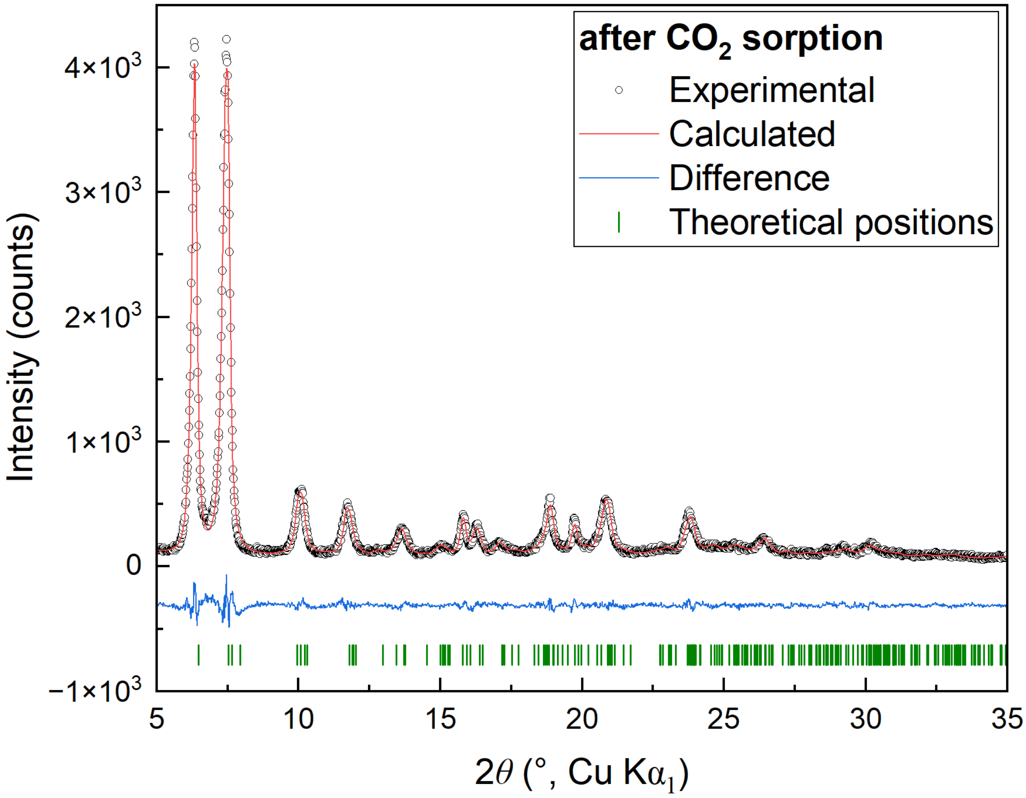

Figure S 20: Rietveld refinement of NTU-9-d after CO_2 gas adsorption.

Table S 4: Refinement data of NTU-9-d (space group $P\bar{1}$) after CO_2 (R_{wp}=7.9) sorption.

	**after CO_2 sorption**
**a/ Å**	13.10(1)
**b/ Å**	15.26(1)
**c/ Å**	11.32(1)
**α/ °**	95.3(1)
**β/ °**	79.0(1)
**γ/ °**	116.6(1)
**V/ Å³**	1988(2)

S4. Flexibility study

S4.1. Resuspension

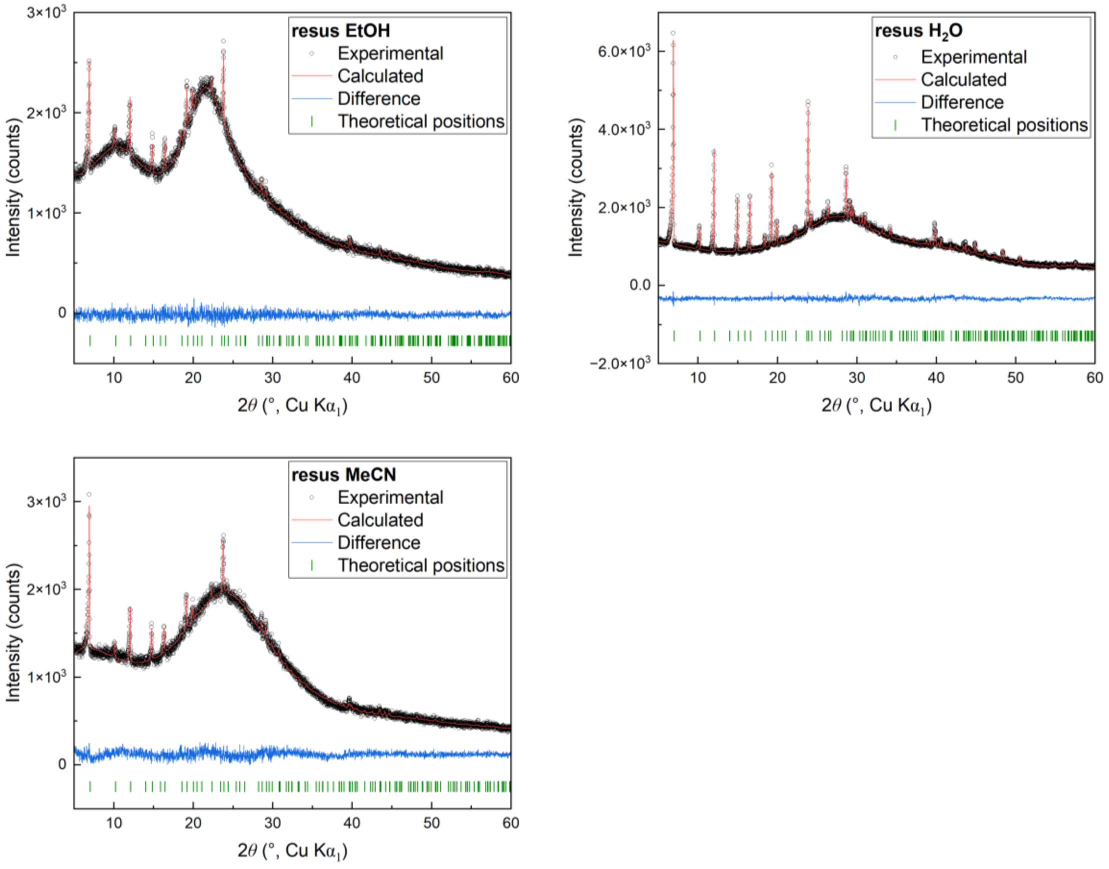

Figure S 21: Rietveld refinements of **S1$_{5d}$** after resuspending NTU-9-d in ethanol, water, and acetonitrile.

Table S 5: Refinement of **S1$_{5d}$** after resuspending NTU-9-d in ethanol, acetonitrile, and water.

	**EtOH**	**MeCN**	**H_2O**
space group	$P\bar{3}1c$	$P\bar{3}1c$	$P\bar{3}1c$
a/Å	14.61(2)	14.60(2)	14.63(2)
c/Å	11.86(2)	11.93(2)	11.77(2)
V/Å^3	2192(2)	2200(2)	2182(2)

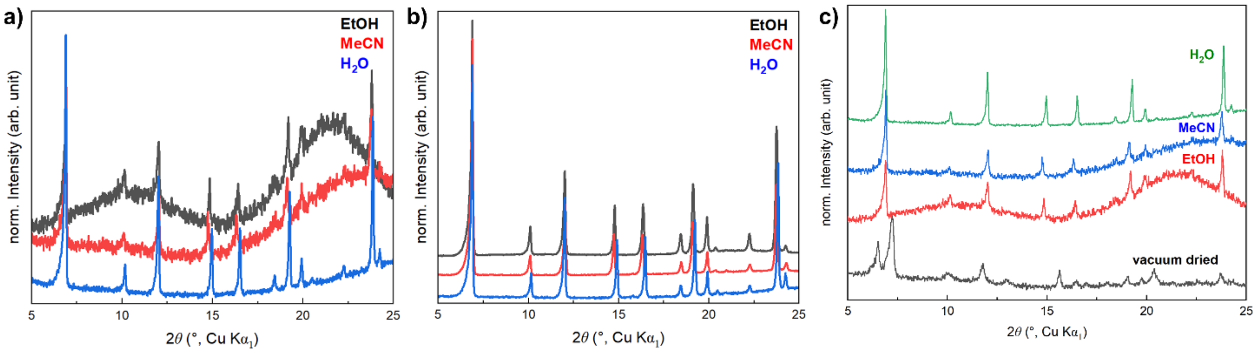

Figure S 22: Overlay of diffraction patterns after resuspension in ethanol, (black), acetonitrile (red), and water (blue) of a) **S1**$_{5d}$ and b) **S2**$_{1d}$. c) Resuspension of NTU-9-d (black) in ethanol (red), acetonitrile (blue), and water (green).

S4.2. Cycles NTU-9 →NTU-9-d

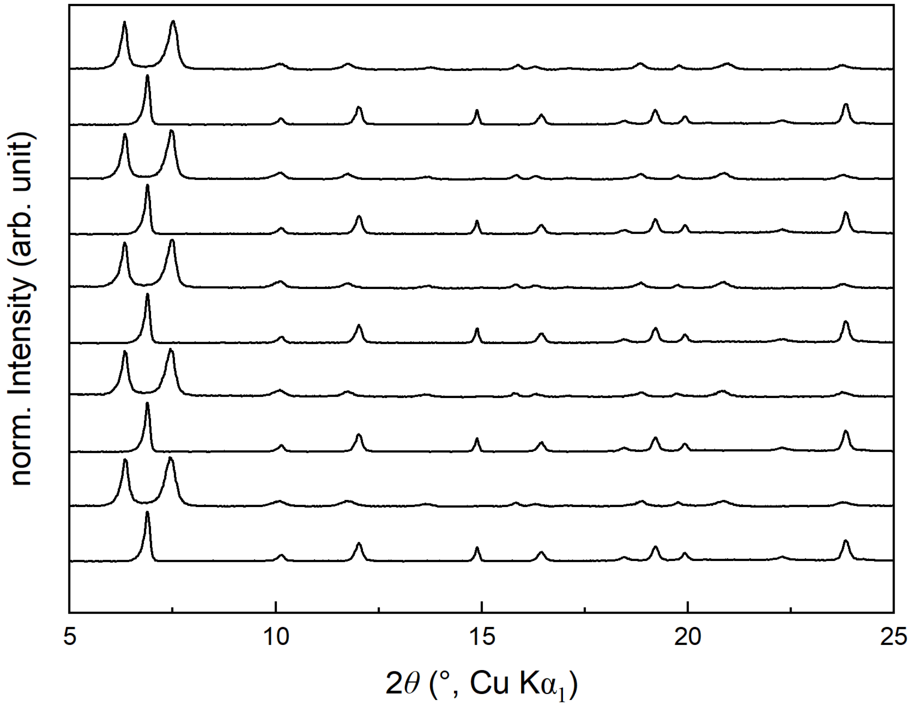

Figure S 23: Five cycles of drying with vacuum at 60 °C and resuspending in EtOH of **S1**.

S6. References

1. J. Gao, J. Miao, P.-Z. Li, W. Y. Teng, L. Yang, Y. Zhao, Bin Liu and Q. Zhang, A p-type Ti(iv)-based metal–organic framework with visible-light photo-response, *Chem. Commun.*, 2014, **50**, 3786-3788.
2. S. Yu, Y. Xiao, Z. Liu, J.-M. Lyu, Y.-L. Wang, Z.-Y. Hu, Y. Li, M. Sun, L.-H. Chen and B.-L. Su, Ti-MOF single-crystals featuring an intracrystal macro–microporous hierarchy for catalytic oxidative desulfurization, *Chem. Commun.*, 2023, **59**, 1801-1804.
3. D. Yan, J. Jia, K. Tong, J. Ouyang and Y. Pan, Metal organic framework as a matrix for laser desorption/ionisation of environmental pollutants and other small molecules, *Int. J. Environ. Ana. Chem.*, 2023, **104**, 9153-9163.
4. M. Brunelli and A. N. Fitch, A glass capillary cell for *in situ* powder X-ray diffraction of condensed volatile compounds. Solid HCFC-123a and HCFC-124, *Journal of Synchrotron Radiation*, 2003, **10**, 337-339.
5. A. A. Coelho, *TOPAS* and *TOPAS-Academic*: an optimization program integrating computer algebra and crystallographic objects written in C++, *J. Appl. Crystallogr.*, 2018, **51**, 210-218.
6. R. W. Cheary and A. Coelho, A fundamental parameters approach to X-ray line-profile fitting, *J. Appl. Crystallogr.*, 1992, **25**, 109-121.
7. A. L. Bail, H. Duroy and J. L. Fourquet, Ab-initio structure determination of $LiSbWO_6$ by X-ray powder diffraction, *Mater. Res. Bull.*, 1988, **23**, 447-452.
8. H. M. Rietveld, A profile refinement method for nuclear and magnetic structures, *J. Appl. Crystallogr.*, 1969, **2**, 65-71.
9. A. A. Coelho, Whole-profile structure solution from powder diffraction data using simulated annealing, *J. Appl. Crystallogr.*, 2000, **33**, 899-908.
10. J. W. M. Osterrieth, J. Rampersad, D. Madden, N. Rampal, L. Skoric, B. Connolly, M. D. Allendorf, V. Stavila, J. L. Snider, R. Ameloot, J. Marreiros, C. Ania, D. Azevedo, E. Vilarrasa-Garcia, B. F. Santos, X.-H. Bu, Z. Chang, H. Bunzen, N. R. Champness, S. L. Griffin, B. Chen, R.-B. Lin, B. Coasne, S. Cohen, J. C. Moreton, Y. J. Colón, L. Chen, R. Clowes, F.-X. Coudert, Y. Cui, B. Hou, D. M. D'Alessandro, P. W. Doheny, M. Dincă, C. Sun, C. Doonan, M. T. Huxley, J. D. Evans, P. Falcaro, R. Ricco, O. Farha, K. B. Idrees, T. Islamoglu, P. Feng, H. Yang, R. S. Forgan, D. Bara, S. Furukawa, E. Sanchez, J. Gascon, S. Telalović, S. K. Ghosh, S. Mukherjee, M. R. Hill, M. M. Sadiq, P. Horcajada, P. Salcedo-Abraira, K. Kaneko, R. Kukobat, J. Kenvin, S. Keskin, S. Kitagawa, K.-i. Otake, R. P. Lively, S. J. A. DeWitt, P. Llewellyn, B. V. Lotsch, S. T. Emmerling, A. M. Pütz, C.

Martí-Gastaldo, N. M. Padial, J. García-Martínez, N. Linares, D. Maspoch, J. A. S. d. Pino, P. Moghadam, R. Oktavian, R. E. Morris, P. S. Wheatley, J. Navarro, C. Petit, D. Danaci, M. J. Rosseinsky, A. P. Katsoulidis, M. Schröder, X. Han, S. Yang, C. Serre, G. Mouchaham, D. S. Sholl, R. Thyagarajan, D. Siderius, R. Q. Snurr, R. B. Goncalves, S. Telfer, S. J. Lee, V. P. Ting, J. L. Rowlandson, T. Uemura, T. Iiyuka, M. A. van derVeen, D. Rega, V. Van Speybroeck, S. M. J. Rogge, A. Lamaire, K. S. Walton, L. W. Bingel, S. Wuttke, J. Andreo, O. Yaghi, B. Zhang, C. T. Yavuz, T. S. Nguyen, F. Zamora, C. Montoro, H. Zhou, A. Kirchon and D. Fairen-Jimenez, How Reproducible are Surface Areas Calculated from the BET Equation?, *Adv. Mater.*, 2022, **34**, 2201502.

11. E. Dhivya, D. Magadevan, Y. Palguna, T. Mishra and N. Aman, Synthesis of titanium based hetero MOF photocatalyst for reduction of Cr (VI) from wastewater, *J. Environ. Chem. Eng.*, 2019, **7**, 103240.
12. Y. Yu, S. Li, L. Huang, J. Yu, H. Zhang, S. Song and T. Zeng, Solar-driven CO_2 conversion promoted by MOF-on-MOF homophase junction, *Catal. Commun.*, 2021, **150**, 106270.
13. L. Zhang and Y. H. Hu, Structure distortion of $Zn_4O_{13}C_{24}H_{12}$ framework (MOF-5), *Mater. Sci. Eng., B*, 2011, **176**, 573-578.
14. C. Chinchilla-Garzón, M. Galbiati, A. Misturini, P. Gimeno-Fonquernie, N. Almora-Barrios, N. M. Padial and C. Martí-Gastaldo, Structural Control of Photoconductivity in a Flexible Titanium-Organic Framework, *Adv. Mater*, 2025, DOI: https://doi.org/10.1002/adma.202412045, 2412045.
15. B. Bueken, F. Vermoortele, D. E. P. Vanpoucke, H. Reinsch, C. C. Tsou, P. Valvekens, T. D. Baerdemaeker, R. Ameloot, C. E. A. Kirschhock, V. V. Speybroeck, J. M. Mayer and D. De Vos, A Flexible Photoactive Titanium Metal–Organic Framework Based on a $[Ti(IV)_3(\mu_3\text{-}O)(O)_2(COO)_6]$ Cluster, *Angew. Chem. Int. Ed.*, 2015, **54**, 13912-13917.
16. T. Loiseau, C. Serre, C. Huguenard, G. Fink, F. Taulelle, M. Henry, T. Bataille and G. Férey, A Rationale for the Large Breathing of the Porous Aluminum Terephthalate (MIL-53) Upon Hydration, *Chem. Eur. J.*, 2004, **10**, 1373-1382.
17. C. Mellot-Draznieks, C. Serre, S. Surblé, N. Audebrand and G. Férey, Very Large Swelling in Hybrid Frameworks: A Combined Computational and Powder Diffraction Study, *J. Am. Chem. Soc.*, 2005, **127**, 16273-16278.
18. C. Serre, C. Mellot-Draznieks, S. Surblé, N. Audebrand, Y. Filinchuk and G. Férey, Role of Solvent-Host Interactions That Lead to Very Large Swelling of Hybrid Frameworks, *Science*, 2007, **315**, 1828-1831.
